# Characterization ofantifungal properties of lipopeptide-producing *Bacillus velezensis* strains and their proteome-based response to the phytopathogens, *Diaporthe* spp

**DOI:** 10.3389/fbioe.2023.1228386

**Published:** 2023-08-07

**Authors:** Stephen Olusanmi Akintayo, Behnoush Hosseini, Maliheh Vahidinasab, Marc Messmer, Jens Pfannstiel, Ute Bertsche, Philipp Hubel, Marius Henkel, Rudolf Hausmann, Ralf T. Voegele, Lars Lilge

**Affiliations:** ^1^ Department of Bioprocess Engineering, Institute of Food Science and Biotechnology, University of Hohenheim, Stuttgart, Germany; ^2^ Department of Phytopathology, Institute of Phytomedicine, University of Hohenheim, Stuttgart, Germany; ^3^ Core Facility Hohenheim, Mass Spectrometry Core Facility, University of Hohenheim, Stuttgart, Germany; ^4^ Cellular Agriculture, TUM School of Life Sciences, Technical University of Munich, Freising, Germany; ^5^ Department of Molecular Genetics, University of Groningen, Groningen, Netherlands

**Keywords:** antifungal, *Bacillus*, biocontrol, lipopeptides, surfactin, phytopathogens, proteomics

## Abstract

**Introduction:** *B. velezensis* strains are of interest in agricultural applications due to their beneficial interactions with plants, notable through their antimicrobial activity. The biocontrol ability of two new lipopeptides-producing *B. velezensis* strains ES1-02 and EFSO2-04, against fungal phytopathogens of *Diaporthe* spp., was evaluated and compared with reference strains QST713 and FZB42. All strains were found to be effective against the plant pathogens, with the new strains showing comparable antifungal activity to QST713 and slightly lower activity than FZB42.

**Methods:** Lipopeptides and their isoforms were identified by high-performance thin-layer chromatography (HPTLC) and mass spectrometric measurements. The associated antifungal influences were determined in direct *in vitro* antagonistic dual culture assays, and the inhibitory growth effects on *Diaporthe* spp. as representatives of phytopathogenic fungi were determined. The effects on bacterial physiology of selected *B. velezensis* strains were analyzed by mass spectrometric proteomic analyses using nano-LC-MS/MS.

**Results and Discussion:** Lipopeptide production analysis revealed that all strains produced surfactin, and one lipopeptide of the iturin family, including bacillomycin L by ES1-02 and EFSO2-04, while QST713 and FZB42 produced iturin A and bacillomycin D, respectively. Fengycin production was however only detected in the reference strains. As a result of co-incubation of strain ES1-02 with the antagonistic phytopathogen *D. longicolla*, an increase in surfactin production of up to 10-fold was observed, making stress induction due to competitors an attractive strategy for surfactin bioproduction. An associated global proteome analysis showed a more detailed overview about the adaptation and response mechanisms of *B. velezensis*, including an increased abundance of proteins associated with the biosynthesis of antimicrobial compounds. Furthermore, higher abundance was determined for proteins associated with oxidative, nitrosative, and general stress response. In contrast, proteins involved in phosphate uptake, amino acid transport, and translation were decreased in abundance. Altogether, this study provides new insights into the physiological adaptation of lipopeptide-producing *B. velezensis* strains, which show the potential for use as biocontrol agents with respect to phytopathogenic fungi.

## 1 Introduction

Plant pathogens pose a threat to food security and agricultural activities due to their ability to cause diseases in economically important crops, leading to low yields and possible crop failure. Phytopathogenic fungi are particularly problematic as they are soilborne and have a diverse host range, making them difficult to control (Summerell et al., 2007; Hazarika et al., 2019). In current agricultural practice, chemical pesticides are widely used to control plant pathogens. However, chemical control agents can have significant impacts on the environment. In addition, the use of chemical fungicides may have other unintended effects, such as development of resistance by pathogens (Ongena and Jacques, 2008; González-Jaramillo et al., 2017). Alternative measures with less environmental impact are therefore desirable for the control of plant pathogens, making microbial agents with antagonistic activity a promising option (Fravel, 2005). In this context, *Bacillus velezensis* strains are of relevance for biocontrol due to their well-documented positive interaction with plants through antimicrobial activities and induction of systemic resistance (Cawoy et al., 2015). In addition, they show plant growth-promoting activities through mechanisms of nitrogen fixation, phosphorus solubilization (Oleńska et al., 2020; Castaldi et al., 2021), regulation of plant hormone production and reduction of abiotic stress through biofilm formation (Bhat et al., 2020). Furthermore, *B. velezensis* produces a variety of potent antimicrobial compounds, including cyclic lipopeptides (LPs). LPs provide direct protection to plants through disease suppression via antibiosis (Meena and Kanwar, 2015) and indirectly through induced systemic resistance (ISR), which acts by stimulation of early immune response and activation of defense mechanisms (Pršić and Ongena, 2020; Tsotetsi et al., 2022).

LPs are broadly classified into three families of surfactin, iturin and fengycin. Structurally, surfactin and iturin are heptapeptides, while fengycin is a decapeptide linked to a fatty acid chain (Ongena and Jacques, 2008). The ribosomally independent biosynthesis of LPs is based on multi-modular mega-enzymes known as non-ribosomal peptide synthetases (NRPSs) or hybrids of polyketide synthases and non-ribosomal peptide synthetases (PKSs-NRPSs) (Walsh, 2014). LPs are often synthesized as a mixture of homologues and isoforms differing in the length of the fatty acid chain and in the composition of the amino acids of the peptide sequences (Płaza et al., 2015; Akintayo et al., 2022). The composition of LPs, the distribution of isoforms, as well as the length of the fatty acid chain could affect their biological activity (Juhaniewicz-Dębin et al., 2020). It is also assumed that an additional effect on the activity is achieved when more than one LP family is present, and when lipopeptides are synthesized alongside other antimicrobial compounds (Lilge et al., 2022). Surfactin has been reported with extensive antibacterial, antifungal and antiviral activity, while iturin and fengycin have been associated mostly with antifungal activity (Ongena and Jacques, 2008; Vahidinasab et al., 2022).

While considerable effort has been devoted to studying the direct antagonistic activity of *Bacillus* strains, there is very little understanding of the broader molecular mechanisms involved in the adaptation, stress response, and offensive strategies adopted by *B. velezensis* when co-localized with fungal phytopathogens. Insight into this important aspect can be gained by investigating the global proteomic response of *B. velezensis* to fungal plant pathogens.

The current study investigates the LP production capacity and antifungal potential of *B. velezensis* strains ES1-02 and EFSO2-04 in comparison with established *B. velezensis* biocontrol strains QST713 and FZB42. The *B. velezensis* strains were used to antagonize *Diaporthe* spp. which are notorious fungal pathogens of economically important crops such as soybeans, sunflowers, grapes, and citrus fruits (Hosseini et al., 2020). The molecular strategies of the promising *B. velezensis* strain ES1-02 to co-localization and antagonism of the phytopathogen *D. longicolla* were examined in greater details via proteomic analysis. In this way, the proteomic adaptation of a *B. velezensis* biocontrol strain in the presence of a phytopathogen provides information on the physiology of *Bacillus* strains for use in agriculture.

## 2 Material and methods

### 2.1 Microorganisms used in the study and cultivation procedures

All bacterial and fungal strains used in this work are described in Supplementary Table S1. The bacterial strains were stored at −80°C using 50% (*v/v*) glycerol. Fungi were maintained on acidified potato dextrose agar plates (APDA) at 10°C and were sub-cultured regularly at room temperature (24°C ± 1°C). The *B. velezensis* isolates ES1-02 and EFSO2-04 are deposited in the National Collection of Industrial Food and Marine Bacteria (NCIMB), UK, with identification numbers NCIMB 15453 and NCIMB 15455, respectively.

### 2.2 Detection of LP biosynthetic genes

Genes encoding LP synthesis, including *srfAA*, *ituB*, *fenA,* and *fenD* were screened by PCR method. The primers used for the PCR reactions, except *ituB* (Dunlap et al., 2019), were designed from the conserved gene region of the genes with information received from multiple sequence alignments of gene sequences obtained from GenBank using Clustal Omega (EMBL-EBI, Hinxton, Cambridge, UK). PCR reaction was performed using PEQSTAR Thermal Cycler (VWR International GmbH, Darmstadt, Germany). The PCR product was purified using a QIAGEN QIAquick PCR Purification kit (QIAGEN AB, Kista, Sweden), and then sequenced (Eurofins Genomics, Ebersberg, Germany). All primers used in this work are listed in Supplementary Table S2.

### 2.3 Liquid chromatography–mass spectrometry (LC–MS) for LP analysis

LC**–**MS analysis of LPs was performed on a 1290 UHPLC system (Agilent, Waldbronn, Germany) coupled to a Q-Exactive Plus Orbitrap mass spectrometer equipped with a heated electrospray ionization (HESI) source (Thermo Fisher Scientific GmbH, Braunschweig, Germany). LPs were separated on an Eclipse C18 column (Agilent, Waldbronn, Germany; 2.1 mm × 50 mm, 1.8 μm particle size). The column temperature was maintained at 40°C. Samples were dissolved in methanol and 5 µL of each sample was injected. Mobile phase A was 0.1% formic acid in water, and mobile phase B was 0.1% formic acid in acetonitrile. A constant flow rate of 0.3 mL/min was used, and gradient elution was performed as follows: 10%–50% B from 1 to 5 min, 50%–95% B from 5 to 15 min, isocratic at 95% B from 15 to 20 min, the system was returned to initial conditions from 95% B to 10% B from 20 to 22 min.

The HESI source was operated in positive ion mode, with a capillary voltage of 4.2 kV and an ion transfer capillary temperature of 360°C. The sheath gas flow rate and auxiliary gas flow rate were set to 60 and 20, respectively. The S-Lens RF level was 50%. The Q-Exactive Plus mass spectrometer was calibrated externally in positive ion mode using the manufacturers calibration solutions (Pierce, Thermo Fisher Scientific GmbH, Braunschweig, Germany). Mass spectra were acquired in MS mode within the mass range of 500–1700 *m/z* at a resolution of 70,000 FWHM (Full Width at Half Maximum) using an Automatic Gain Control (AGC) target of 1.0 × 10^6^ and 100 ms maximum ion injection time. Data dependent MS/MS spectra in a mass range of 200–2000 *m/z* were generated for the five most abundant precursor ions with a resolution of 17,500 FWHM using an AGC target of 3.0 × 10^6^ and 120 ms maximum ion injection time and a stepped collision energy of 15, 30 and 45. Xcalibur™ software version 4.3.73 (Thermo Fisher Scientific, San Jose, United States) was used for data acquisition and data analysis. Commercial standards of surfactin, fengycin and iturin A (all from Sigma-Aldrich, Seelze, Germany) were used as reference for LP identification in *B. velezensis* strains.

### 2.4 Cultivation of *B. velezensis* strains in mineral salt medium (MSM)

For preparation of pre-cultures, 100 µL of a glycerol stock was inoculated into 10 mL of LB medium (5 g/L tryptone, 10 g/L NaCl, 10 g/L yeast extract) in 100 mL baffled shake flasks and cultivated overnight at 37 °C and 120 rpm using a shaking incubator (Newbrunswick™/Innova^®^ 44, Eppendorf AG, Hamburg, Germany). Subsequently, second precultures prepared with 25 mL mineral salt medium (MSM) in 250 mL shake flasks were inoculated with the first precultures to a starting optical density (OD_600_) of 0.1 and incubated for approx. 16 h. The main cultivation was carried out in 50 mL MSM in 500 mL shake flasks representing a 10% filling volume which has been demonstrated to provide sufficient oxygen availability for an aerated bioprocess (Hoffmann et al., 2021). The main cultivations were inoculated with the second precultures to a starting OD_600_ of 0.1. MSM was prepared according to Willenbacher et al. (2015) with slight modification. The medium contained 8 g/L glucose, 4.0 µM Na_2_EDTA × 2 H_2_O, 7 µM CaCl_2_, 4 µM FeSO_4_, 1 µM MnSO_4_, 50 mM Urea, 30 mM KH_2_PO_4_, 40 mM Na_2_HPO_4_ and 800 µM MgSO_4_. All cultivations were carried out in three biological replicates at 37°C and 0.4 *g* (orbit of 5.1 cm, shaking frequency of 120 rpm) in an incubation shaker (Innova 44®R, Eppendorf AG). Samples were taken at regular interval and the OD_600_ measured using a spectrophotometer (WPA CO8000, Biochrom Ltf., Cambridge, UK). The samples were subsequently centrifuged at 7,164 *g* and 4°C for 10 min and cells were removed. The supernatants were stored at −20°C until further processing.

### 2.5 Extraction of LPs and sample analysis by high-performance thin-layer chromatography (HPTLC)

LPs were extracted according to Geissler et al. (2019). In brief, a threefold extraction of 2 mL of cell-free broth with 2 mL of a mixture of chloroform/methanol (2:1; *v/v*) was conducted. The pooled solvent layers were evaporated to dryness via a rotary evaporator (RVC2-25 Cdplus, Martin Christ Gefriertrocknungsanlagen GmbH, Osterode am Harz, Germany) at 10 mbar and 40°C. LPs were analyzed with an HPTLC system (CAMAG, Muttenz, Switzerland) according to a method described by Geissler et al. (2019) with little modification. In detail, dried samples were re-suspended in 2 mL of methanol and applied as 6 mm bands on HPTLC silica gel 60 plates (Merck, Darmstadt, Germany). A two-step development was performed for the simultaneous quantification of surfactin, iturin/bacillomycin and fengycin. The first development was performed with a mixture of chloroform/methanol/water (65:25:4; *v/v/v*) as the mobile phase, and a developing distance of 60 mm, while the second development was carried out using the mobile phase butanol/ethanol/0.1% acetic acid (1:4:1; *v/v/v*) over a migration distance of 60 mm as well. To detect and quantify surfactin and iturin/bacillomycin, plates were scanned after the first development, while fengycin was quantified after second development. All LPs were scanned at UV 195 nm. Surfactin, iturin and fengycin standards (Sigma-Aldrich, St. Louis, United States) were used for calibration.

### 2.6 Physiological data analysis

Prior to data analysis, OD_600_/cell dry weight (CDW) conversion factor was determined as described by (Geissler et al., 2019). CDW, glucose concentration, and LP (surfactin, iturin or bacillomycin, and fengycin) titers at sampling time point were plotted. The yield of biomass per substrate Y_X/S_ (g/g), product per biomass Y_P/S_ (g/g), growth rate (μ), and specific productivity q_P/X_ (g_product_/g_CDW_ h) were determined with the equations below. Y_P/S_, Y_P/X_ and q_P/X_ were determined at the maximum LPs (surfactin, iturin, bacillomycin or fengycin [P_max_]) concentrations, while Y_X/S_ was determined at the maximum CDW. ΔS is the change in substrate between the time point at which a value was calculated and the beginning of cultivation (t_0_). Δt is change in time (h) between the point of calculated value and t_0_. The Y_X/S_ was determined at the maximum CDW, while Y_P/S_ and Y_P/X_ were analyzed at the maximum product concentration. For the calculation of growth rate, the time intervals between the start of cultivation and the time point of highest CDW during the exponential phase were used. The following analyses were done with equations reported by (Klausmann et al., 2021).

$$Y_{X/S}=\frac{X}{\Delta S}$$ (1)

$$Y_{P/S}=\frac{Pmax}{\Delta S}$$ (2)

$$Y_{P/X}=\frac{Pmax}{X}$$ (3)

$$q_{P/X}=\frac{Pmax}{Xpmax\;*\;\Delta t}$$ (4)

$$µ=\frac{\ln\left(x2\right)-\ln\left(x1\right)}{\Delta t}$$ (5)

### 2.7 Direct *in vitro* antagonistic assay (dual culture)

Antagonistic ability of each *B. velezensis* strain against *Diaporthe* spp. was evaluated using the dual culture technique. Therefore, a *B. velezensis* strain was inoculated at one side of an APDA plate and a 6 mm disc of an actively growing *Diaporthe* sp. was inoculated on the opposite side of the plate and incubated at 25°C. *Diaporthe* spp. inoculated on one side of the plate without co-inoculation of *B. velezensis* strains were used as control. All experiments were done in three biological replicates. Fungal growth was measured from day 5 to day 7. To determine the effects on *Diaporthe* spp. morphology caused by co-incubation with *B. velezensis* strains, mycelial samples taken from regions next to the inhibition zone were examined under light microscope. The mycelia taken from the edge of the growing fungal in the control plates were also examined.

Antifungal activity was expressed as percentage growth inhibition and was calculated with the following equation.

$$Inhibition=\left[\frac{Rc\;–\;Ri}{Rc}\right]*100\%$$ (6)

In this context, *R*_*i*_ is the distance (mm) from the point of inoculation towards the edge of the colony in dual culture, and *R*_*c*_ is the corresponding distance (mm) of mycelium determined in the control.

### 2.8 Detection of LP concentration in APDA plates

Agar plugs of approximately 500 mg were taken from the inhibition zones using a cork borer. To de-structure the agar and prepare for homogenization, agar plugs were frozen overnight at −20°C. Subsequently, 1 mL sterile distilled water was added to the agar and homogenized with a mechanical homogenizer (MICCRA MiniBatch D-9, MICCRA GmbH, Heitersheim, Germany) at 11,000 rpm for 2 min and afterwards mixed thoroughly by vortexing. Subsequently, LPs were extracted and quantitatively measured by HPTLC.

### 2.9 Sample preparation for proteome analysis

*B. velezensis* strain ES1-02 was co-cultivated with *D. longicolla* DPC_HOH20 on APDA plates as previously described. A control experiment was set up with ES1-02 cultivated alone on APDA plates. The experiments were set up in seven biological replicates. After 5 days of cultivation with a visible zone of inhibition between bacteria and fungi, the edge of the bacterial colony next to the inhibition zone was gently scraped with a sterile loop and immediately put in 40 µL lysis buffer (2% SDS, 50 mM Tris-HCl pH 6.8, 20 mM DTT). In the control experiment, samples were collected at the edge of the growing ES1-02 colonies after 5 days of cultivation and put into lysis buffer.

### 2.10 Protein extraction, on-bead digest, and peptide purification for proteome analysis

Bacterial samples in lysis buffer were subsequently heated for 5 min at 95°C and 700 rpm in a ThermoMixer^®^ (Eppendorf ThermoMixer^®^ C, Hamburg, Germany). After centrifugation at 20,000 g for 5 min, protein concentrations of the cleared supernatants were determined by the Bradford assay (Bradford, 1976) and adjusted to a protein concentration of 1 μg/μL using Tris-HCl (pH 8.5) and lysis buffer. In total, 28 µL of cleared lysate was reduced and alkylated (10 mM Tris-(2-Carboxyethyl)phosphine (TCEP), 40 mM chloroacetamide (CAA) for 30 min at room temperature in the dark and proteins were subsequently extracted by single-pot solid-phase-enhanced sample preparation (SP3; 1:1 mixture of SpeedBeads™ magnetic carboxylate modified particles 50 mg/mL; Cytiva; CAT No: 45152105050250 and 65152105050250) (Hughes et al., 2014). A volume of 10 µL of activated SP3 Bead-Mix and ethanol to a final concentration of 80% was added to the sample and incubated for 5 min at room temperature. Samples were mounted on a magnetic rack and briefly washed three times with 80% ethanol in 50 mM Tris/HCl (pH 8.5).

Proteins were digested on the SP3 beads in 0.2% sodium-deoxycholate (SDC), 50 mM ammonium bicarbonate using trypsin (Roche, Penzberg, Germany) and LysC (FUJIFILM Wako Pure Chemical Corporation, Osaka, Japan) in a 1:100 and 1:200 protein to protease ratio, respectively. The protein digest was performed for 20 h at 36°C and 800 rpm. The digest was stopped by adding formic acid (FA) and acetonitrile (ACN) to the samples at final concentrations of 0.5% FA and 96% ACN. Samples were washed on a magnetic rack three times with 98% ACN and 0.05% FA. Peptides were eluted from the beads with water and lyophilized in a vacuum concentrator (Concentrator plus, Eppendorf, Hamburg, Germany). Peptides were solubilized in 0.1% trifluoroacetic acid (TFA) prior to LC-MS/MS analysis.

### 2.11 Nano-LC-MS/MS proteome analysis

Nano-LC-ESI-MS/MS experiments were performed on an Ultimate 3,000 nano-RSLC (Thermo Fisher Scientific, Germering, Germany) coupled to an Exploris 480 mass spectrometer (Thermo Fisher Scientific, Bremen, Germany) using a Nanospray-Flex ion source (Thermo Fisher Scientific, Bremen, Germany). Peptides were concentrated and desalted on a trap column (5 mm × 30 μm, Thermo Fisher Scientific, Dreieich, Germany) and separated on a 25 cm × 75 µm nanoEase MZ HSS T3 reversed phase column (100 Å pore size, 1.8 µm particle size, Waters, Milforf, MA, United States of America) operated at constant temperature of 35°C. Peptides were separated at a flow rate of 300 nL/min using a gradient with the following profile: 2%–8% solvent B for 4 min, 8%–16% solvent B for 22 min, 16%–30% solvent B for 11 min, 30%–95% solvent B for 4 min, isocratic 95% solvent B for 4 min and 95%–2% solvent B for 5 min. Solvents used were 0.1% FA (solvent A) and 0.1% FA in ACN/H_2_O (80/20, *v/v*, solvent B).

MS spectra (*m/z* = 300–1,500) were detected in the Orbitrap at a resolution of 60,000 (*m/z* = 200). Maximum injection time (MIT) for MS spectra was set to 50 ms, the AGC value was set to 3 × 10^6^. Internal calibration of the Orbitrap analyzer was performed using lock-mass ions from ambient air as described by Olsen et al. (2005).

The MS was operating in data dependent mode selecting the top 25 highest abundant peptide precursor signals for fragmentation (HCD, normalized collision energy of 30). For MS/MS analysis only undetermined charge states and charge states from 2-5 of considered; the monoisotopic precursor selection was set to peptides and the minimum intensity threshold was set to 7.5 × 10^4^. MS/MS scans were performed in the Orbitrap at a resolution of 15,000, isolation width was set to 1.6 Da. The AGC target was set to 7 × 10^4^, a maximum injection time (MIT) of 70 ms and a fixed first mass of 120 *m/z*. Dynamic exclusion was set to 60 s with a tolerance of 10 ppm (parts per million).

### 2.12 MS data analysis and protein quantification

Raw files were first processed with MaxQuant (Cox and Mann, 2008). Protein identification in MaxQuant was performed using the integrated database search engine Andromeda (Cox et al., 2011). MS/MS spectra were searched against the *B. velenzensis* protein sequence database downloaded from UniProt (Bateman, 2019). Reversed sequences as decoy database and common contaminant sequences were added automatically by MaxQuant. Mass tolerances of 4.5 ppm for MS spectra and 20 ppm for MS/MS spectra were used. Trypsin was specified as enzyme and two missed cleavages were allowed. Carbamidomethylation of cysteine was set as a fixed modification and protein N-terminal acetylation and methionine oxidation were allowed as variable modifications. The “match between runs” feature of MaxQuant was enabled with a match time window of 1 minute and an alignment time window of 20 min. Peptide false discovery rate (FDR) and protein FDR thresholds were set to 0.01. Protein quantification in MaxQuant was performed by label free quantification (LFQ) with a LFQ minimum ratio count setting of one.

In a second data processing step, MaxQuant result files were fed into the IceR R-package, for further re-quantification using default settings (Kalxdorf et al., 2021). The pre-imputed IceR output file containing LFQ intensities was further normalized according to the median LFQ intensity of the sample and used subsequently for differential expression analysis.

Welch´s t-test, principal component analysis (PCA), hierarchical clustering and Volcano plots were performed using Perseus version 1.6.14.0 (Tyanova et al., 2016). Matches to contaminants (e.g., keratins, trypsin) and reverse databases identified by MaxQuant were excluded from further analysis. First, normalized LFQ values were log2 transformed. Only protein groups with at least 40% valid values in data set were considered for the statistical analysis. Remaining missing values were imputed by the “Replace missing values from normal distribution” function (Width = 0.3, Down shift = 1.8) implemented in Perseus drawing random numbers from a normal distribution representing low abundance intensity values. Significant changes in protein abundance were analyzed by a Welch’s t-test (two sided, S0 = 1) and corrected for multiple hypothesis testing using permutation-based FDR statistics (FDR = 0.05, 250 permutations). For subsequent hierarchical cluster analysis of the significant differential expressed proteins, imputed LFQ intensity values were removed. The mass spectrometry proteomics data was deposited to the ProteomeXchange Consortium via the PRIDE (Perez-Riverol et al., 2022) partner repository with the dataset identifier PXD035016.

## 3 Results

### 3.1 Screening for genes encoding LP biosynthesis

Since *B. velezensis* strains exhibit capability for producing a great range of LPs, the genetic potential of *B. velezensis* strains ES1-02 and EFSO2-04 was compared with reference strains QST713 and FZB42. Specifically, *srfAA* genes from the *srfA* operon encoding the surfactin-forming NRPS were amplified in all strains (Supplementary Table S3). Futhermore, since *B. velezensis* strains are capable of producing different variants of iturin-type LPs, the strains were screened for specific genes encoding different LPs of the iturin class, such as iturin A, bacillomycin L, and bacllomycin D. Therefore, a PCR technique targeting iturin gene (*ituB*) was used to predict the strain-specific nature of the iturin family of LP produced by each strain (Dunlap et al., 2019). In brief, amino acids in positions 1 to 3 of the peptide sequence within the LP are conserved in all iturin families, while amino acids at positions 4 to 7 may be variable (Dunlap et al., 2019). In the PCR approach, the binding site for a forward primer was located in the conserved third NRPS module (third amino acid: AA#3), which allowed integration of the D-asparagine for all three types of iturin (iturin A, bacillomycin D and L). In contrast, the binding sites for the reverse primers were different for each of iturin A, bacillomycin D and bacillomycin L and were located on variable AA#4 modules (iturin A–Gln, bacillomycin D–Pro, bacillomycin L–Ser). In this way, predictions were made about which type of the iturin family is encoded by each strain. In summary, *ituB* genes corresponding to bacillomycin L were detected in the isolates ES1-02 and EFSO2-04, while genes specifically encoding iturin A and bacillomycin D were amplified in reference strains QST713 and FZB42, respectively (Supplementary Table S3). With respect to fengycin, two genes (*fenA* and *fenD*) were targeted for amplification in the *fenABCDE* operon. While *fenA* was amplified in all strains, the amplification of the *fenD* gene was observed only in QST713 and FZB42.

### 3.2 Identification of LPs and their isoforms

While the detection of genes encoding for the production of LPs provides information about the genetic potential of strains, the production of these molecules needs to be determined by reliable methods such as mass spectrometric (MS) analyses. This provides information on the composition of the isoforms (congeners) and the length of the fatty acid chains, which are important properties for biological activities (Henry et al., 2011). The MS results showed that at least two LP types were co-synthesized in each strain (Table 1).

**TABLE 1:** Mass spectrometric identification of LPs and their isoforms.

Strain	Surfactin	Iturin family of LP	Fengycin
ES1-02	Surfactin (C_12_–C_17_)	Bacillomycin L (C_13_–C_17_)	-
EFSO2-04	Surfactin (C_12_–C_17_)	Bacillomycin L (C_13_–C_17_)	-
QST713	Surfactin (C_12_–C_17_)	Iturin A (C_12_–C_17_)	Fengycin A (C_14_–C_18_)
			Fengycin B (C_14_–C_18_)
			Fengycin X (C_15_–C_18_)
			Fengycin Y (C_15_)
FZB42	Surfactin (C_12_–C_17_)	Bacillomycin D (C_11_–C_17_)	Fengycin A (C_14_–C_18_)
			Fengycin B (C_15_–C_18_)
			Fengycin X (C_16_–C_18_)
			Fengycin Y (C_15_)

Further details on the LP detection showed that surfactin, composed of a mixture of C_12_ to C_17_ isoforms, was produced by all four *B. velezensis* strains (Table 1) with the most abundant isoforms being C_14_ for both reference strains and C_15_ for the isolates, respectively, and C_12_ as the least abundant in all strains (Supplementary Figure S1A). However, the iturin family LPs produced by the strains differed in type and isoform composition. In detail, strains ES1-02 and EFSO2-04 produced bacillomycin L with fatty acid chain lengths from C_13_ to C_17_, while QST713 synthesised iturin A with C_12_ to C_17_, and FZB42 produced bacillomycin D isoforms from C_11_ to C_17_ (Table 1). Although the *B. velezensis* strains showed different iturin production, C_15_ and C_13_ were the most and the least abundant isoforms in all strains, respectively (Supplementary Figure S1B). Fengycin synthesis was not detected in the isolates ES1-02 and EFSO3-04, possibly due to shortened versions of the *fen* operon as indicated by the non-detection of the *fenD* genes (Supplementary Table S3). However, reference strains QST713 and FZB42 synthesized fengycin groups A, B, X and Y (Table 1) with varying isoform abundances (Supplementary Figure S1C, D).

### 3.3 Growth behavior and quantification of LPs

**FIGURE 1:**
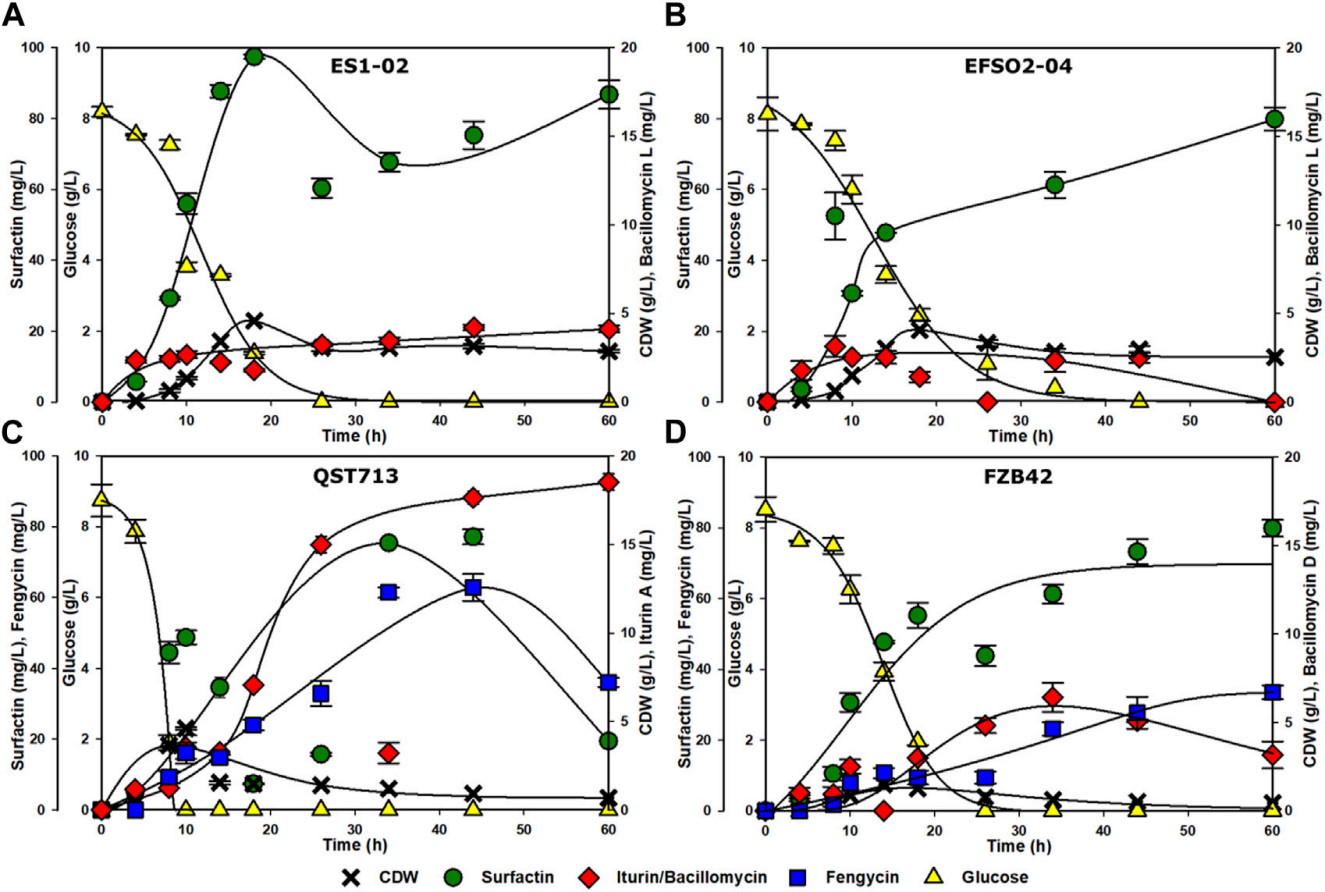
Time courses of the strains ES1-02 **(A)**, EFSO2-04 **(B)**, QST713 **(C)** and FZB42 **(D)** were monitored for CDW (black crosses), glucose consumption (yellow triangle) and production of surfactin (green dots), iturin/bacillomycin (red diamonds) and fengycin (blue squares).

In order to get insights about both the cell growth and LP production of the different *B. velezensis* strains, shake flask cultivations were performed for 60 h at an initial glucose concentration of 8 g/L. ES1-02 and EFSO2-04 reached maximum CDW values of 4.6 and 4.1 g/L, respectively, after 18 h, while QST713 reached comparable maximum CDW values of 4.6 g/L after a shorter cultivation time of 10 h. Strain FZB42, however, achieved a lower maximum CDW of 1.5 g/L after 14 h (Figure 1). Strains ES1-02, EFS02-04, and FZB42 showed growth limitation before glucose was completely consumed, indicating possible additional substrate limitation aside from glucose. Growth limitations associated with nitrogen, phosphate or trace element limitation have been reported in *Bacillus* (Mahmood, 1972; Hu et al., 1999; Hoffmann et al., 2021), and as such the impact on lipopeptide production should be a subject of future investigation.

Regarding LP production, a surfactin titer of 97.4 mg/L after 18 h was measured for ES1-02, while EFSO2-04 produced an equal amount of surfactin as FZB42 after at 79.9 mg/L after 60 h. QST713 however produced 77.2 mg/L after 44 h. The highest amount of bacillomycin L was detected in ES1-02 (4.2 mg/L) after 44 h, while the highest amounts of iturin A and bacillomycin D were achieved in QST713 (18.5 mg/L) and FZB42 (6.4 mg/L) after 60 and 34 h, respectively. Fengycin biosynthesis by QST713 and FZB42 reached maximum of 62.8 after 44 h and 33.5 mg/L and 60 h, respectively.

To properly understand the performance of the strains, several data analyses were performed based on cell growth and LP production and the results summarized in Table 2.

**TABLE 2:** Overview of strains’ performance and lipopeptides production.

Strain	LPs	P_max_ (mg/L)	TimeP_max_ (h)	Y_P/X_ (g/g)	Y_P/S_ (g/g)	q_P/X_ (g g^-1^ h^-1^)	Y_X/S_ (g/g)	μ (h/1)	CDW_max_ (g/L)
ES1-02	Surfactin	97.4	18.0	0.02117	0.01218	0.00118	0.58	0.08	4.60
	Bacillomycin L	4.2	44.0	0.00135	0.00053	0.00003			
EFSO2-04	Surfactin	79.9	66.0	0.03196	0.00999	0.00053	0.59	0.08	4.10
	Bacillomycin L	3.1	8.0	0.00517	0.00517	0.00065			
QST713	Surfactin	77.2	44.0	0.08578	0.00965	0.00195	0.58	0.15	4.60
	Iturin A	18.5	60.0	0.02643	0.00231	0.00044			
	Fengycin	62.8	44.0	0.06978	0.00785	0.00159			
FZB42	Surfactin	79.9	60.0	0.19975	0.00999	0.00333	0.25	0.03	1.50
	Bacillomycin D	6.4	34.0	0.01067	0.00080	0.00031			
	Fengycin	33.5	60.0	0.08375	0.00419	0.00140			

Specifically, ES1-02 and EFSO2-04 showed comparable specific growth rates of 0.08 h^-1^, while a higher specific growth rate of 0.15 h^-1^ was determined for QST713 and a lower rate of 0.03 h^-1^ for FZB42 (Table 2). Biomass yields on substrate Y_x/s_ were similar for ES1-02, EFSO2-04 and QST713. Similarly, ES1-02 showed a higher yield of surfactin on substrate (0.012 g/g) compared to QST713 (0.010 g/g) and FZB42 (0.010 g/g) but was inferior in terms of iturin/bacillomycin yield on substrate. With regards to specific productivities, higher surfactin values were recorded for QST713 and FZB42, while EFSO2-O4 was superior in bacillomycin/iturin.

### 3.4 Antifungal activity of *B. velezensis* strains against phytopathogens *Diaporthe* spp.

**FIGURE 2:**
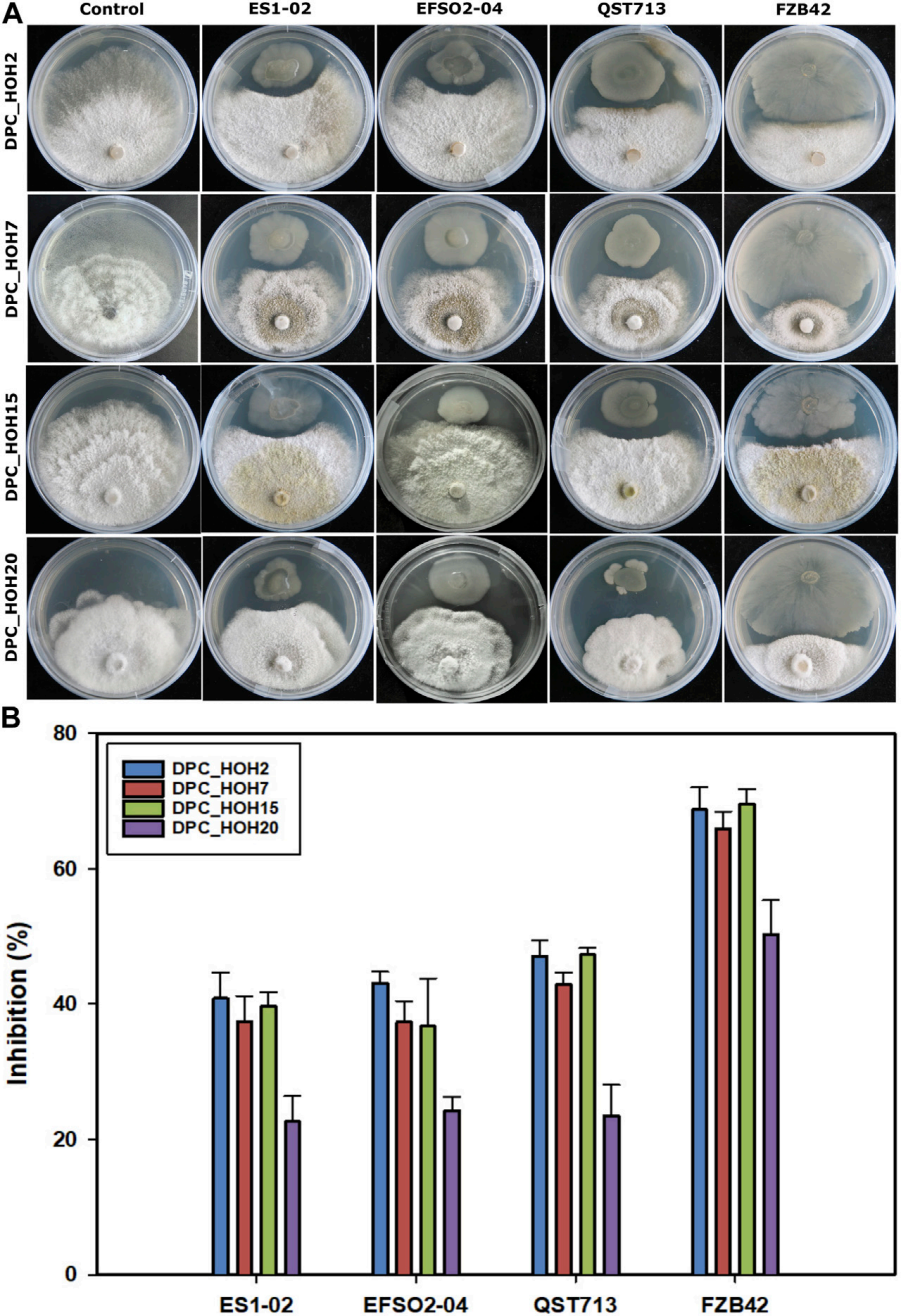
Antagonistic activities of *B. velezensis* strains against *Diaporthe* spp. **(A)** Dual culture assay after 7 days of incubation **(B)** percentage inhibition of *Diaporthe* spp. indicator strains (DPC_HOH2, DPC_HOH7, DPC_HOH15, and DPC_HOH20) by *B. velezensis* strains ES1-02, EFSO2-04, QST713, and FZB42.

Since the *B. velezensis* strains produced different LP combinations with varying amounts and relative isoform abundances, the applicability in terms of antifungal activity against phytopathogens was investigated. For this purpose, the soybean pathogens *Diaporthe* spp. were used as indicator strains. Using the dual *in vitro* antagonistic assay, distinct zones of inhibition were observed between the *B. velezensis* strains and the fungal pathogens *Diaporthe* spp. after 7 days of co-incubation at room temperature (Figure 2A).

In detail, *B. velezensis* strains ES1-02 and EFSO2-04 showed inhibitory efficiencies comparable to the commercial biocontrol strain QST713 with about 40% of inhibition against three of the test *Diaporthe* spp. (DPC_HOH2, DPC_HOH7 and DPC_HOH15). (Figure 2B). In contrast, higher inhibition activities were determined for strain FZB42 against all *Diaporthe* spp. compared to the other *B. velezensis* strains against the indicator pathogens (68.9% DPC_HOH2, 66% DPC_HOH7, 69.6% DPC_HOH15% and 50.4% DPC_HOH20). In more detail, strains ES1-01 and EFSO3-04 were the most effective against DPC_HOH2, while QST713 showed the best inhibitory effect against DPC_HOH2 and DPC_HOH15. Strain FZB42, however, demonstrated the highest efficacy against DPC_HOH15 (Figure 2B).

### 3.5 Effects on the morphology of *Diaporthe* spp.

**FIGURE 3:**
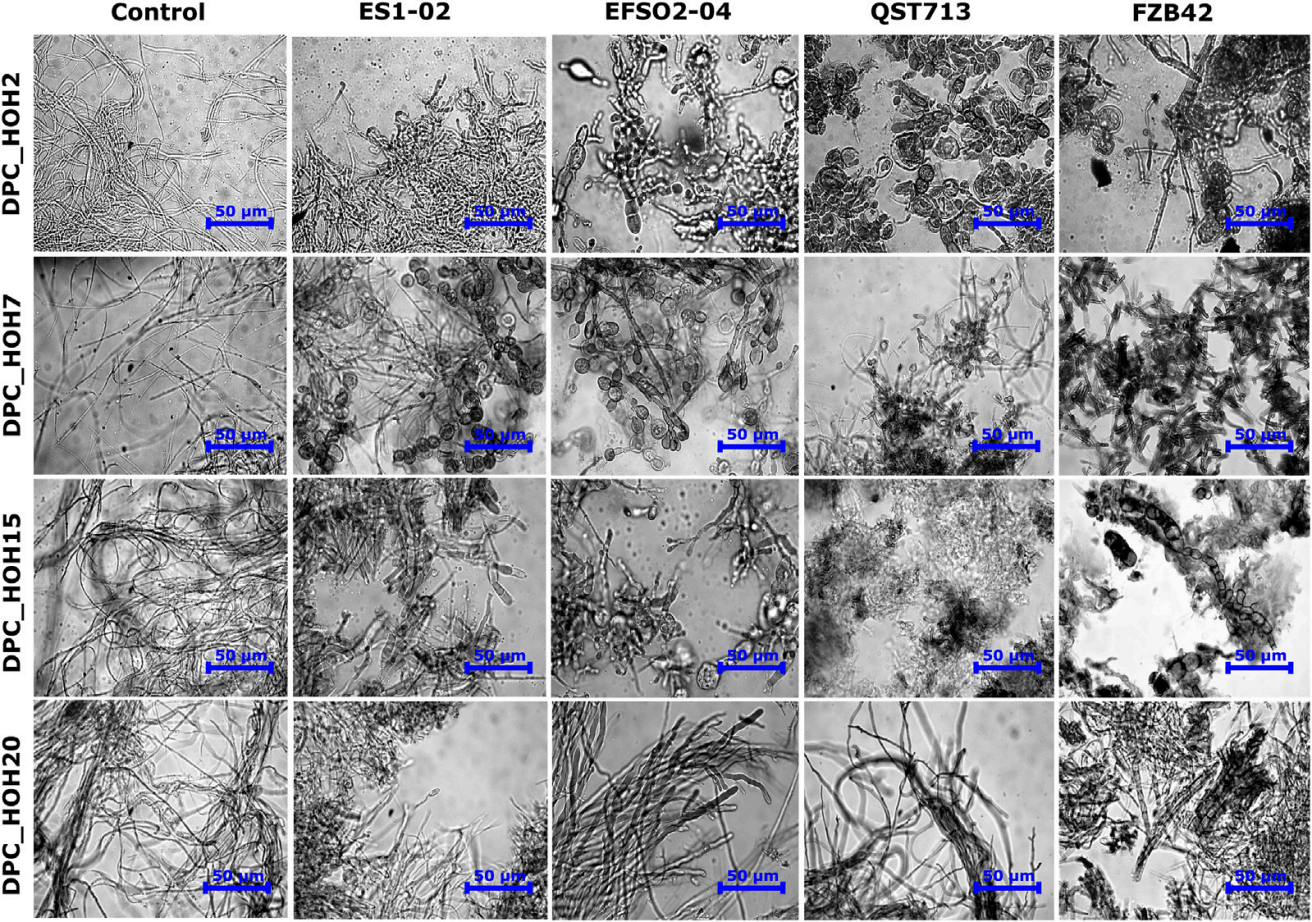
Microscopic images showing the effects of co-incubation with *B. velezensis* strains ES1-02, EFSO2-04, QST713 and FZB42 on the mycelial structures of the *Diaporthe* spp. indicator strains after 7 days of incubation.

In order to evaluate the effects of the *B. velezensis* strains on the fungal pathogens, microscopic examination of the fungal mycelia at the edges of the inhibition zones was carried out. Morphological anomalies such as enlargement of hyphae, swellings, formation of bulbs, or complete disruption of mycelium were observed (Figure 3). In contrast, the mycelium from the control experiments, in which the fungi were not exposed to a *B. velezensis* strain, showed well-developed hyphae (Figure 3). In particular, swollen structures resembling vesicles were observed in indicator pathogens DPC_HOH7 and DPC_HOH2 exposed to *B. velezensis* isolates ES1-02 and EFSO2-04 as well as exposed to reference strain QST713, respectively (Figure 3). The co-cultivation approaches in this study thus show that an antifungal effect is already present under native cultivation conditions and that no purification of the LPs is necessary. This confirms the assumption that both reference strains FZB42 and QST713 as well as the *B. velezensis* isolates ES1-02 and EFSO2-04 presented here can have a beneficial effect in agriculture.

### 3.6 LP induction by the presence of *D. longicolla*

**FIGURE 4:**
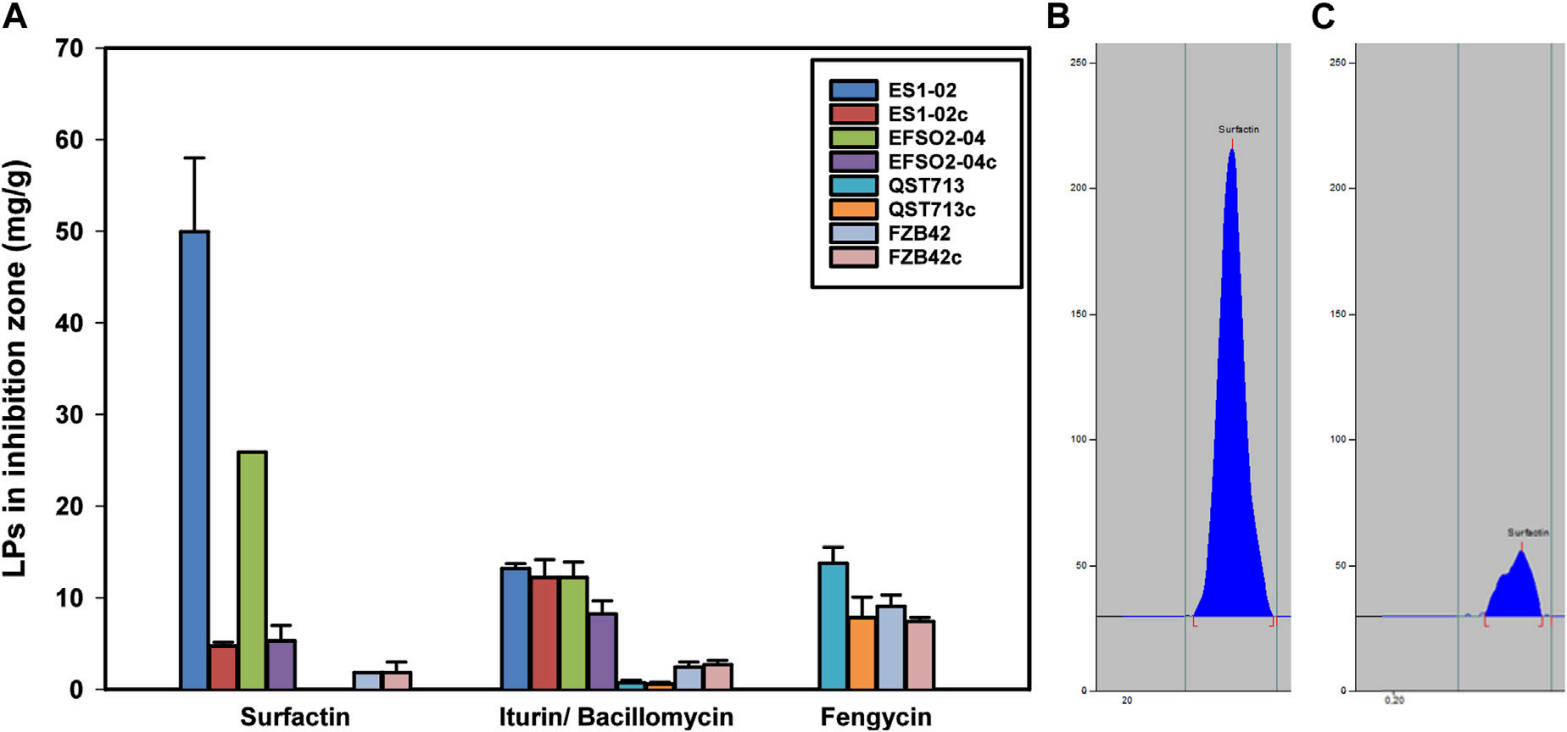
**(A)** Concentrations of surfactin, iturin/bacillomycin and fengycin determined in the zone of inhibition between *B. velezensis* strains ES1-02, EFSO2-04, QST713, and FZB42 co-cultured in presence or absence of *D. longicolla* indicator strain DPC_HOH20 (c - control). **(B)** HPTLC chromatogram of surfactin in ES1-02 co-incubated with *D. longicolla* DPC_HOH20, and **(C)** ES1-02 incubated alone. All quantifications were done in three biological replicates.

Since lipopeptides produced by *Bacillus* strains have been described as versatile weapons for biocontrol of plant diseases (Ongena and Jacques, 2008), further insights into LP production in the presence of pathogens are needed. Accordingly, the amounts of LPs in the inhibition zones were quantified. Therefore, the influence of the interaction of *D. longicolla* (DPC_HOH20) with the *B. velezensis* strains on the respective LP synthesis was determined. In this context, the surfactin accumulation in the inhibition zone of the isolates ES1-02 and EFSO2-04 showed 10-fold and 5-fold increase, respectively, compared to the control experiments, in which the *B. velezensis* strains were cultivated alone (Figure 4). Notably, no increase in surfactin accumulation by reference strains FZB42 and QST713 was observed when co-cultivated with the pathogenic indicator strain DPC_HOH20. Since the highest increase in surfactin accumulation in the inhibition zone was observed in ES1-02, the distribution of surfactin isoforms in the inhibition zone of ES1-02 was also examined and it showed increases in the relative abundances of C_16_ and C_17_ isoforms from 63% to 5% when ES1-02 was cultivated alone to 75% and 9% respectively in the inhibition zone when co-incubated with the *D. longicolla* indicator strain. A decrease in C_13_ isoforms from 10% to 5% was also observed, while the abundances of the other isoforms remain relatively unchanged (Supplementary Figure S2).

In addition, a meaningful increase in bacillomycin L was determined in EFSO2-04, although to a lesser extent than in surfactin, by 1.5-fold (Figure 4). In contrast, the accumulation of bacillomycin D in the inhibition zone of FZB42 was slightly decreased compared to the strain cultivated alone. For fengycin, increased synthesis was detected in both QST713 and FZB42, with the higher induction of 1.5-fold recorded in QST713.

### 3.7 Analysis of the proteome adaptation of *B. velezensis* ES1-02 in presence of *D. longicolla*

**FIGURE 5:**
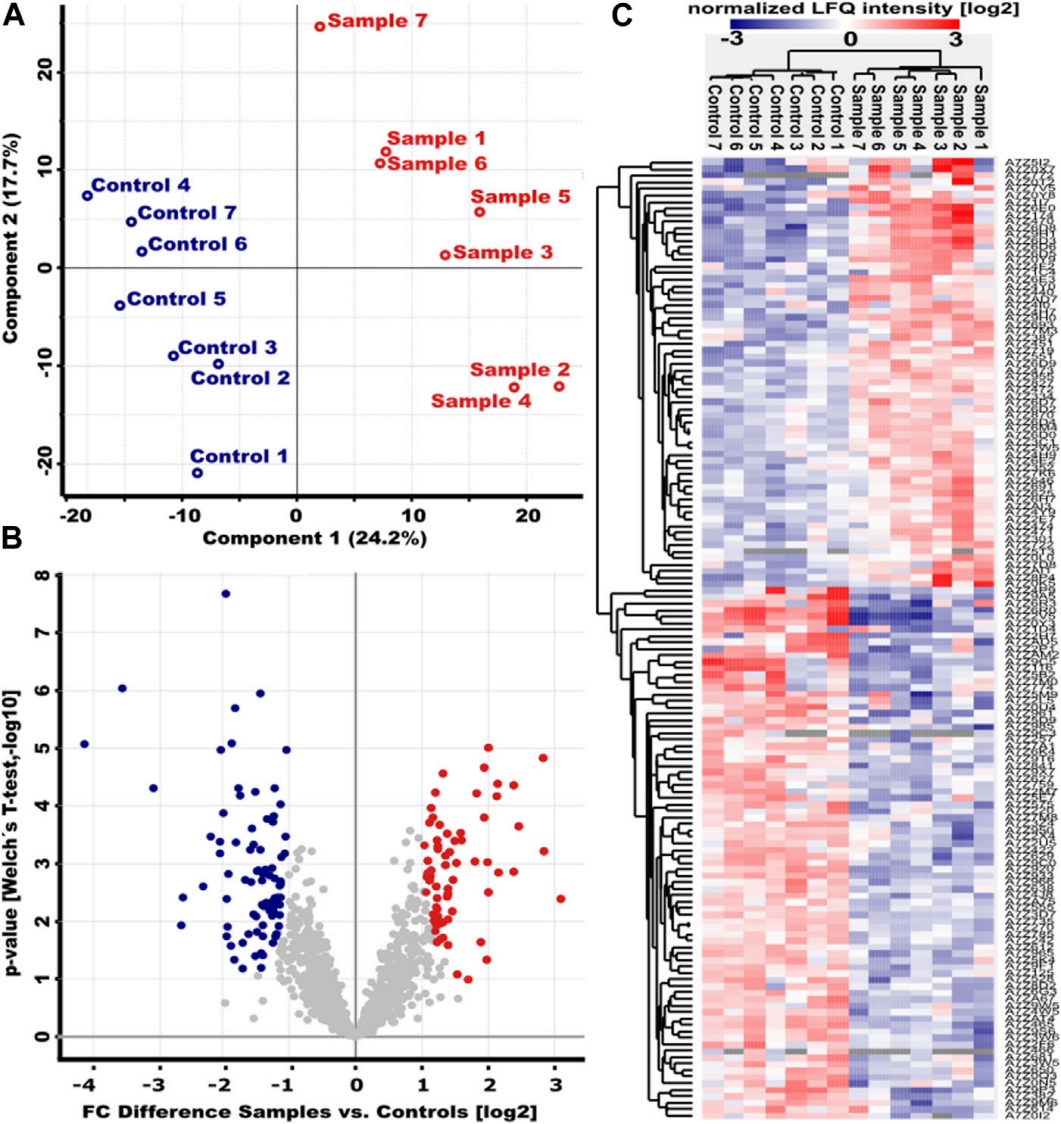
Altered protein abundances in *B. velezensis* strain ES1-02 in the presence of *D. longicolla* DPC_HOH20. **(A)** Principal Component Analysis (PCA) of the ES1-02 strain co-cultivated with *D. longiclolla* (red, sample) and the control experiment (blue, control). Seven biological replicates were analyzed for each of the two conditions. **(B)** Volcano plot of the log10 *p*-values versus the log2 differences in protein abundance between ES1-02 co-cultivated with *D. longicolla* DPC_HOH20 and control. Proteins whose abundance was significantly increased or decreased according to Welch’s t-test (cut-off permutation-based FDR 0.05, S0 = 1) are shown in red and blue, respectively. **(C)** Unsupervised hierarchical cluster analysis of the 148 differentially abundant proteins (log2 normalized LFQ intensities) between samples and controls.

Surfactin has been previously described as a potent biosurfactant with various biological activities (Meena and Kanwar, 2015). Although its antimicrobial properties seem to occur mainly in the presence of synergistic metabolites (Lilge et al., 2022), the amount of surfactin indicates the biocontrol potential of a *Bacillus* strain. Accordingly, the *B. velezensis* strain ES1-02 with a 10-fold induction of extracellular surfactin accumulation during co-cultivation with the pathogenic indicator strain DPC_HOH20 (Figure 4) makes the strain interesting for a more detailed characterization. Accordingly, the bacterial adaptation was analyzed in more detail at the proteome level. To gain more insight into the molecular mechanisms involved in the response of *B. velezensis* to *D. logicolla*, proteome analyses were performed on strain ES1-02 cultured in the presence and absence of DPC_HOH20, respectively. Here, 100% quantification was achieved for 1,215 proteins with IceR (Supplementary Table S4). Principal Component Analysis (PCA) showed a clear separation between ES1-02 co-cultivated with *D. longiclolla* DPC_HOH20 and control experiments (Figure 5A).

A total of 148 (66 with higher abundance, 82 lower with abundance) proteins were found with significantly altered abundance between absence and presence of DPC_HOH20 (Figures 5B,C). A closer look at the protein abundances showed that about 23% of all proteins with significantly higher abundance associated with the production of bioactive secondary metabolites such as bacillaenes and polyketides (PRIDE dataset identifier PXD035016), for which extensive antimicrobial activities have been reported (Alenezi et al., 2021). In addition, about 11% of the proteins were found to be involved in the intracellular *Bacillus* oxidative, nitrosative and general stress response, as well as DNA repair, leading to non-specific, multiple and preventive resistance (Hecker and Völker, 2001). Interestingly, several proteins involved in fatty acid biosynthesis and modification (12%) were also found with higher abundance. This is reasonable in that fatty acid composition varies in response to environmental conditions and is important for cellular adaptation and might be a defence strategy regarding antagonistic co-cultivation (Diomandé et al., 2015; Joo et al., 2016).

In contrast, several proteins associated with the provision of phosphate (7%) and modification of the cell surface (7%), such as peptidoglycan-binding proteins and biosynthesis of teichoic/teichuronic acids and phospholipids, were detected at lower abundance. Interestingly, proteins associated with Fe-S cluster or binding these cluster were additionally determined with a lower abundance. Since, as described above, evidence for oxidative stress by correspondingly induced stress proteins could be detected, it can be assumed that this stress reaction is linked to a reduced biosynthesis of proteins with Fe-S clusters. In this way, defective proteins mediated by oxidized Fe-S clusters are avoided and further formation of radicals mediated by the Fenton reaction is reduced (Roche et al., 2013). In addition, proteins involved in amino acid import and biosynthesis (e.g., histidine, arginine and glutamate) (10%) and translation (10%) were detected with lower abundance, suggesting that *B. velezensis* cells localized close to phytopathogens reduce their metabolism. Table 3 summarizes the proteins that were most affected by co-cultivation with DPC_HOH20.

**TABLE 3:** Overview of *B. velezensis* ES1-02 proteins showing the most altered abundance due to co-cultivation with *D. longicolla* DPC_HOH20.

UniProt ID	Log2 difference	Protein name/description	Function, biological process, and biosynthetic pathway
Induced			
A7Z772	3.10	Sporulation protein, YhcN/YlaJ-like protein	sporulation-associated protein
A7Z174	2.84	Nitrite reductase (large subunit)	nitrate reduction (assimilation)
A7Z1I7	2.83	Uncharacterized protein	Unknown
A7Z6D3	2.46	SDR family NAD(P)-dependent oxidoreductase	involved in bacillaene biosynthesis; catalytic activity and small molecule binding (phosphopantetheine binding)
A7Z6E0	2.38	AMP-binding protein	O-succinylbenzoate-CoA ligase; menaquinone biosynthesis
A7Z6D8	2.38	SDR family NAD(P)-dependent oxidoreductase	involved in bacillaene biosynthesis; lipid metabolism
A7Z8P4	2.16	Cupin domain-containing protein	oxalate decarboxylase
A7Z9H1	2.14	Acetolactate synthase AlsS	overflow metabolism; acetoin metabolism
A7Z0Y9	2.13	Uncharacterized protein	putative N-terminal signal peptide
A7Z470	2.01	[Acyl-carrier-protein] S-malonyltransferase	putative FabD protein; fatty acid biosynthesis
A7Z6D5	2.00	SDR family NAD(P)-dependent oxidoreductase	involved in bacillaene biosynthesis; fatty acid biosynthesis
A7Z6D6	1.99	SDR family NAD(P)-dependent oxidoreductase	properly involved in bacillaene biosynthesis; fatty acid biosynthesis
Repressed			
A7Z629	−1.99	Transcription attenuation protein MtrB	regulation of tryptophan biosynthesis and translation; attenuation in the *trp* operon; repression of the folate operon
A7Z465	−2.03	DNA-directed RNA polymerase subunit epsilon	control of RNA polymerase activity
A7Z9P3	−2.05	VWA domain-containing protein	Unknown
A7Z961	−2.05	Histidine biosynthesis bifunctional protein HisIE	biosynthesis of histidine
A7ZAM2	−2.19	Uncharacterized protein	VWA domain-containing protein
A7Z116	−2.29	Alkaline phosphatase	putative PhoD protein; acquisition of phosphate upon phosphate starvation; degradation of wall teichoic acid during phosphate starvation
A7Z9C5	−2.60	Uncharacterized protein	putative TuaF protein; biosynthesis of teichuronic acid
A7Z9A5	−2.63	Flagellin	putative Hag protein; flagellin protein; motility and chemotaxis
A7Z0Y3	−3.10	Glycerophosphodiester phosphodiesterase	similar to GlpQ protein; glycerol-3-phosphate utilization; degradation of wall teichoic acid during phosphate starvation
A7Z6R0	−3.57	Phosphate import ATP-binding protein PstB	high-affinity phosphate uptake
A7Z2V5	−4.13	Alkaline phosphatase	putative PhoA protein; alkaline phosphatase A; acquisition of phosphate upon phosphate starvation

## 4 Discussion

Crop efficiency in agriculture is directly related to plant health. In this context, plant-associated pathogens impair crop productivity, resulting in lower yields and crop losses. Accordingly, the use of wild-type bacterial strains with high ability to biosynthesize products with antifungal properties is useful as biocontrol agents. In particular, *B. velezensis* strains have been widely described for the production of various LPs, which have been associated with antibacterial and antifungal activities. Moreover, the efficacy of LPs could be improved when different LP families are present, leading to an additive effect (Lilge et al., 2022). In this study, two novel *B. velezensis* strains, ES1-02 and EFSO2-04, were compared with the already established biocontrol reference strains QST713 and FZB42 in terms of their LP production and antifungal activity. To gain a more comprehensive understanding of the bacterial response to fungal interaction, the microbial adaptations of *B. velezensis* to co-localization and antagonism to the phytopathogen were examined at the proteome level.

Interestingly, both ES1-02 and EFSO2-04 produced bacillomycin L, while iturin A and bacillomycin D were identified for reference strains QST713 and FZB42, respectively (Table 1). The main variability among iturin class of LP is in the amino acids at positions 4 to 7 in their heptapeptide sequences. While bacillomycin L has Ser, Glu, Ser, Thr at the positions 4 to 7, bacillomycin D has Pro, Glu, Ser, Thr, and iturin A has Gln, Pro, Asn, Ser (Dunlap et al., 2019). Here, the different amino acid composition could influence the antimicrobial activity within the iturin LP family as amino acid sequence as been reported as one of the factors influencing LP biological activity (Oliveras et al., 2021). In contrast, only the reference strains showed fengycin biosynthesis. In this context, the present work is the first report on the synthesis of fengycin X and Y by strains QST713 (Supplementary Figures S15–S17) and FZB42 (Supplementary Figures S22–S24), which were first identified by Ait Kaki et al. (2020) in the *B. amyloliquefaciens* (ET) strain. The simultaneous production of multiple fengycin variants by QST713 and FZB42 has important consequences for their status as biocontrol agents, as extensive antifungal activities have been documented for fengycin (Anastassiadou et al., 2021). The non-production of fengycin by ES1-02 and EFSO2-04 can be explained on the basis of incomplete fengycin operons. Since LP-forming NRPSs are encoded by operons consisting of several genes, the absence of one or more genes in the operon could lead to the strain being unable to synthesize the product. In particular, the synthesis of fengycin is encoded by the *fenABCDE* operon (Tosato et al., 1997), meaning that a missing *fenD* gene as observed in ES1-02 and EFSO2-04, would result in the strains being unable to produce fengycin (Lin et al., 2005). Occurrence of missing genes in the fengycin biosynthetic gene cluster has been reported to be fairly common among members of the *B. velezensis* clade (Steinke et al., 2021), corroborating the non-detection of the *fenD* gene in ES1-02 and EFSO2-04.

A number of factors may influence the type, amount, and variability of LP isoforms produced by *Bacillus* strains. These factors include their genetic capacity, the composition of the cultivation medium, and the cultivation conditions (Dunlap et al., 2019; Lilge et al., 2021). Exemplarily, since growth limitation of the isolates ES1-02, EFS02-04, and reference strain FZB42 was determined before glucose was depleted as the sole carbon source, additional substrate limitations (e.g., nitrogen, phosphate, and trace elements) may be present that affect lipopeptide production and isoform formation. In particular, fatty acid variation appears to have an impact on LP activity. Accordingly, the bioactivity of LPs with longer acyl chains may be due to the ability of these LPs to be fully incorporated into membranes, while shorter fatty acid chains may not span the membrane, causing less membrane disruption (Maget-Dana and Ptak, 1995). In addition, LPs with longer fatty acid chains may form oligomers that increase the permeability of fungal membranes, enhancing their antifungal activity (Malina and Shai, 2005). In this way, Henry et al. (2011) reported that surfactin isoforms with lipid chains of C_14_ and C_15_ induced a significant immune response in cells of tobacco plants, while homologues with C_12_ and C_13_ showed no activity. The authors suggested that C_14_ and C_15_ surfactin probably represent most of the activity in the surfactin mixture. This indicates that the surfactin isoform mixtures of ES1-02 and EFSO2-04 may be more active than those of QST713 and FZB42 due to the higher abundance of surfactin isoforms with longer fatty acid chains. In addition, higher relative abundances of surfactin isoforms C_16_ and C_17_ in the inhibition zone of ES1-02 compared to when the strain was not confronting a fungal pathogen suggested that the production of increased amount of surfactin isoforms with longer fatty acid chain as a possible strategy for antifungal activity. Similarly (Tabbene et al., 2011), showed that the antimicrobial activity of bacillomycin D-like LP against *Candida* was increased with increasing fatty acid chain length. Increased antifungal activity of bacillomycin L with longer fatty acid chain has also been similarly reported (Eshita et al., 1995). While the relative abundance of the C_15_ bacillomycin/iturin isoform was highest in all strains in this study, it is noticeable that strain FZB42 produced the C_17_ and C_16_ isoforms in higher relative abundances than the other strains (Fig. S1). These isoforms possibly contributed to the higher antifungal activity of the strain.

In addition to the study of microbial LP productivity and characterization of LP isoforms, antifungal activities were also analyzed using the soybean pathogens *Diaporthe* spp. as indicator strains. Here, *B. velezensis* isolates ES1-02 and EFSO2-04 showed antifungal activity comparable to that of reference strain QST713. However, FZB42 showed about 20% higher inhibitory activity, which may be partly due to relatively rapid colonization of the agar (Figure 2). To understand the molecular background for this improved swarming ability, an examination of the genome of FZB42 from the NCBI database revealed the presence of several genes, such as *swrA*, *hag*, *motA*, and *motB*, which encode proteins for both motility and chemotaxis. In contrast, the reference strain QST713 encodes the same genes but has significantly less colonization. Comparing these proteins required for motility (accession numbers CP025079 and CP000560), the identity between the reference strains is 98% for SwrA, 95% for Hag, 100% for MotA, but only 30% for MotB. Accordingly, the relatively high difference in MotB protein sequence (flagellar stator subunit) could be a reason for the reduced motility of QST713. Similar reasons might also be present for the *B. velezensis* isolates ES1-02 and EFSO2-04.

Nevertheless, significant changes in *Diaporthe* spp. morphology were observed as a result of co-cultivation with all *B. velezensis* strains, including swellings, formation of bulbs or complete disruption of mycelium (Figure 3). Desmyttere et al. (2019) reported similar morphological damage in the form of swollen structures upon exposure of various fungal pathogens to crude LP mixtures as well as isolated fengycin. Hyphal swelling was reported in *Rhizopus stolonifer* when exposed to fengycin (Tang et al., 2014). In addition, Romero et al. (2007) reported bulb formation in mycelia of *P. fusca* when its conidia were treated with purified fengycin. They also documented membrane disruption in *P. fusca* conidia when exposed to purified bacillomycin or iturin. While the exact molecular mechanism for the antimicrobial activity of LPs is still the subject of research, several authors have described membrane damage, which can occur through disruption or pore formation, as the primary mode of action of LPs against fungal pathogens (Romero et al., 2007; Kulimushi et al., 2017).

Although the *B. velezensis* strains showed antifungal activity, the presence of phytopathogens associated with the biosynthesis of extracellular molecules could be a stress factor for the cells. Therefore, in a first step, the changes in extracellular LP accumulation were analyzed. While surfactin and, with a lower inducible effect, fengycin were identified with higher amounts, LP accumulations of the bacillomycin/iturin family showed only minor variations, suggesting that surfactin in particular is involved in the extracellular stress and antagonistic response of *B. velezensis* during co-presence with the phytopathogenic fungi (Figure 4). Accordingly, the overall LP productivity of *B. velezensis* might be higher in nature due to antagonistic interactions with other microbes. A previous report by Kulimushi et al. (2017) on the interaction of *B. velezensis* strains S499, FZB42, and QST713 with *Rhizomucor variabilis*, a fungal pathogen of maize, showed 10-fold and 5.2-fold induction of fengycin synthesis by S499 and FZB42, respectively, whereas no induction was reported for QST713. The study additionally reported no induction of surfactin or iturin synthesis by all three strains. Another study also documented increased synthesis of bacillomycin and fengycin when *B. amyloliquefaciens* SQR9 was challenged with *F. oxysporum*, but when faced with *Rhizoctonia solani* and *F. solani*, surfactin was induced while fengycin production decreased (Li et al., 2014). In this context, several molecular regulatory systems are involved that have antagonistic effects on the expression of the different LPs-forming NRPSs (Lilge et al., 2021). Thus, overall, LP inducibility in response to fungal phytopathogens appears to be strain-specific. In particular, since *B. velezensis* strains ES1-02 and EFSO2-04 showed greater surfactin accumulation in the presence of the phytopathogenic indicator strain *D. longicolla* DPC_HOH20 (Figure 4), the lipopeptide family of surfactins seems to be notably involved in the extracellular stress response in the co-presence of antagonistic microbes. Here, the bioactive properties of surfactin, which cause permeabilization of biomembranes, could be useful as a kind of peptide antibiotic in microbial communities (Carrillo et al., 2003). This observation is reasonable because the general stress response of *Bacillus*, controlled by the alternative sigma factor B, plays a positive role in surfactin bioproduction (Bartolini et al., 2019). Since the other lipopeptide families showed only relatively small changes in their abundances during co-cultivation with the indicator strain, and strain ES1-02 in particular showed a strong induction of surfactin amounts, the physiological adaptation of *B. velezensis* ES1-02 was analyzed in more detail (Figure 5). In order to detect changes in protein abundance, samples were taken from cells of the ES1-02 strain located immediately at the edge of the colony at the closest possible distance from the DPC_HOH20 indicator strain to ensure the greatest possible impact during co-cultivation. However, since sampling was performed after 5 days of cultivation to ensure a growth inhibitory effect on the indicator strain, and the method of sampling was associated with some variance in the amount of material, a total of 7 biological replicates were used to ensure significance in the results regarding differences in protein abundances between control experiments and cocultures in the comparative MS-based proteome analyses. During co-cultivation, an increased abundance of proteins related to the biosynthesis of bioactive molecules, such as bacillaenes and polyketides, was measured. In addition, higher levels of oxidative, nitrosative, and general stress response proteins were determined, suggesting both an intracellular defense and repair strategy and an extracellular offensive strategy. Furthermore, *B. velezensis* appears to rearrange the fatty acid availability during co-localization with phytopathogens. Thus, in addition to a possible impact on membrane fluidity, the putatively increased fatty acid synthesis could have a positive effect on the surfactin biosynthesis. As already mentioned, fatty acid biosynthesis is critically involved in surfactin synthesis and plays important roles in determining its activity and properties (Théatre et al., 2021). On the other hand, several proteins associated with phosphate recruitment and cell surface modification were reduced in abundance, suggesting cell surface reorganization. In addition, proteins involved in the biosynthesis and transport of amino acids, such as for arginine, histidine, and glutamate, and in translation were reduced. In addition, proteins having functionality mediated by Fe-S clusters were found to be reduced. Since *B. velezensis* cells are assumed to be exposed to oxidative stress based on the identification of corresponding stress proteins, a molecular strategy to reduce Fe-S cluster-containing proteins is reasonable. In this way, oxidative damage leading to nonfunctional proteins and the formation of radicals by the Fenton reaction could be minimized.

## 5 Conclusion

The results of this study show that the *B. velezensis* strains ES1-02 and EFSO2-04 are effective biocontrol strains with overall promising LP production yields and comparable antifungal activity to the reference strain QST713. Remarkably, co-localization of *B. velezensis* and the phytopathogen *D. longicolla* DPC_HOH20 increased the extracellular surfactin accumulation in ES1-02 and EFSO2-04. In addition, more detailed proteomic analyses revealed microbial adaptation in response to co-localization with phytopathogens, including increased abundance of proteins related to the biosynthesis of antimicrobial compounds such as polyketides and a decrease in proteins involved in phosphate provision, translation and amino acid synthesis. Accordingly, stress conditions induced by phytopathogens could help to enhance the beneficial properties of *B. velezensis* that characterize it as a biocontrol strain.

## Data Availability

The datasets presented in this study can be found in online repositories. The names of the repository/repositories and accession number(s) can be found in the article/Supplementary Material.

## References

[B1] Ait Kaki A. Smargiasso N. Ongena M. Kara Ali M. Moula N. de Pauw E. (2020). Characterization of new Fengycin cyclic lipopeptide variants produced by *Bacillus amyloliquefaciens* (ET) originating from a Salt Lake of Eastern Algeria. Curr. Microbiol. 77 (3), 443–451. 10.1007/S00284-019-01855-W 31894376

[B2] Akintayo S. O. Treinen C. Vahidinasab M. Pfannstiel J. Bertsche U. Fadahunsi I. (2022). Exploration of surfactin production by newly isolated *Bacillus* and *Lysinibacillus* strains from food related sources. Lett. Appl. Microbiol. 2, 378–387. 10.1111/LAM.13731 35486075

[B3] Alenezi F. N. Slama H. B. Bouket A. C. Cherif-Silini H. Silini A. Luptakova L. (2021). *Bacillus velezensis*: A treasure house of bioactive compounds of medicinal, biocontrol and environmental importance. Forests 12 (12), 1714. 10.3390/f12121714

[B4] Anastassiadou M. Arena M. Auteri D. Brancato A. Bura L. Carrasco Cabrera L. (2021). Peer review of the pesticide risk assessment of the active substance *Bacillus amyloliquefaciens* strain QST 713 (formerly *Bacillus subtilis* strain QST 713). EFSA J. 19 (1), e06381. 10.2903/J.EFSA.2021.6381 33519992 PMC7818626

[B5] Bartolini M. Cogliati S. Vileta D. Bauman C. Ramirez W. Grau R. (2019). Stress-responsive alternative sigma factor SigB plays a positive role in the antifungal proficiency of *Bacillus subtilis* . Appl. Environ. Microbiol. 85 (9), 001788–e219. 10.1128/AEM.00178-19 PMC6495766 30824454

[B6] Bateman A. (2019). UniProt: A worldwide hub of protein knowledge. Nucleic Acids Res. 47 (D1), D506–D515. 10.1093/NAR/GKY1049 30395287 PMC6323992

[B7] Bhat M. A. Kumar V. Bhat M. A. Wani I. A. Dar F. L. Farooq I. (2020). Mechanistic insights of the interaction of plant growth-promoting rhizobacteria (PGPR) with plant roots toward enhancing plant productivity by alleviating salinity stress. Front. Microbiol. 11, 1952. 10.3389/FMICB.2020.01952 32973708 PMC7468593

[B9] Bradford M. M. (1976). A rapid and sensitive method for the quantitation of microgram quantities of protein utilizing the principle of protein-dye binding. Anal. Biochem. 72 (1–2), 248–254. 10.1016/0003-2697(76)90527-3 942051

[B10] Carrillo C. Teruel J. A. Aranda F. J. Ortiz A. (2003). Molecular mechanism of membrane permeabilization by the peptide antibiotic surfactin. Biochimica Biophysica acta 11 (1–2), 91–97. 10.1016/s0005-2736(03)00029-4 12659949

[B11] Castaldi S. Petrillo C. Donadio G. Piaz F. D. Cimmino A. Masi M. (2021). Plant growth promotion function of *Bacillus* sp. strains isolated from salt-Pan rhizosphere and their biocontrol potential against *Macrophomina phaseolina* . Int. J. Mol. Sci. 22 (7), 3324. 10.3390/IJMS22073324 33805133 PMC8036593

[B12] Cawoy H. Debois D. Franzil L. de Pauw E. Thonart P. Ongena M. (2015). Lipopeptides as main ingredients for inhibition of fungal phytopathogens by *Bacillus subtilis/amyloliquefaciens* . Microb. Biotechnol. 8 (2), 281–295. 10.1111/1751-7915.12238 25529983 PMC4353342

[B14] Cox J. Mann M. (2008). MaxQuant enables high peptide identification rates, individualized p.p.b. range mass accuracies and proteome-wide protein quantification. Nat. Biotechnol. 26 (12), 1367–1372. 10.1038/nbt.1511 19029910

[B15] Cox J. Neuhauser N. Michalski A. Scheltema R. A. Olsen J. v. Mann M. (2011). Andromeda: A peptide search engine integrated into the MaxQuant environment. J. Proteome Res. 10 (4), 1794–1805. 10.1021/pr101065j 21254760

[B16] Desmyttere H. Deweer C. Muchembled J. Sahmer K. Jacquin J. Coutte F. (2019). Antifungal activities of *Bacillus subtilis* lipopeptides to two *Venturia inaequalis* strains possessing different tebuconazole sensitivity. Front. Microbiol. 10, 2327. 10.3389/fmicb.2019.02327 31695685 PMC6817503

[B17] Diomandé S. E. Nguyen-The C. Guinebretière M. H. Broussolle V. Brillard J. (2015). Role of fatty acids in *Bacillus* environmental adaptation. Front. Microbiol. 6, 813. 10.3389/fmicb.2015.00813 26300876 PMC4525379

[B18] Dunlap C. A. Bowman M. J. Rooney A. P. (2019). Iturinic lipopeptide diversity in the *Bacillus subtilis* species group – important antifungals for plant disease biocontrol applications. Front. Microbiol. 10, 1794. 10.3389/fmicb.2019.01794 31440222 PMC6693446

[B70] Eshita S. M. Roberto N. H. Beale J. M. Mamiya B. M. Workman R. F. (1995). Bacillomycin Lc, a new antibiotic of the iturin group: isolations, structures, and antifungal activities of the congeners. J. Antibiot. 48 (11), 1240–1247. 10.7164/antibiotics.48.1240 8557563

[B21] Fravel D. R. (2005). Commercialization and implementation of biocontrol. Annu. Rev. 43, 337–359. 10.1146/Annurev.Phyto.43.032904.092924 16078888

[B22] Geissler M. Kühle I. Morabbi Heravi K. Altenbuchner J. Henkel M. Hausmann R. (2019). Evaluation of surfactin synthesis in a genome reduced *Bacillus subtilis* strain. Amb. Express 9 (1), 84. 10.1186/S13568-019-0806-5 31190306 PMC6562014

[B23] González-Jaramillo L. M. Aranda F. J. Teruel J. A. Villegas-Escobar V. Ortiz A. (2017). Antimycotic activity of fengycin C biosurfactant and its interaction with phosphatidylcholine model membranes. Colloids Surfaces B Biointerfaces 156, 114–122. 10.1016/J.COLSURFB.2017.05.021 28527355

[B24] Hazarika D. J. Goswami G. Gautom T. Parveen A. Das P. Barooah M. (2019). Lipopeptide mediated biocontrol activity of endophytic *Bacillus subtilis* against fungal phytopathogens. BMC Microbiol. 19 (1), 71–13. 10.1186/S12866-019-1440-8 30940070 PMC6444643

[B25] Hecker M. Völker U. (2001). General stress response of *Bacillus subtilis* and other bacteria. Adv. Microb. Physiology 44, 35–91. 10.1016/S0065-2911(01)44011-2 11407115

[B26] Henry G. Deleu M. Jourdan E. Thonart P. Ongena M. (2011). The bacterial lipopeptide surfactin targets the lipid fraction of the plant plasma membrane to trigger immune-related defence responses. Cell. Microbiol. 13 (11), 1824–1837. 10.1111/J.1462-5822.2011.01664.X 21838773

[B27] Hoffmann M. Braig A. Fernandez Cano Luna D. S. Rief K. Becker P. Treinen C. (2021). Evaluation of an oxygen-dependent self-inducible surfactin synthesis in *B. subtilis* by substitution of native promoter P_srfA_ by anaerobically acitve P_narG_ and P_nasD_ . Amb. Express 11 (1), 57. 10.1186/s13568-021-01218-4 33876328 PMC8055807

[B28] Hosseini B. El-Hasan A. Link T. Voegele R. T. (2020). Analysis of the species spectrum of the *Diaporthe/Phomopsis* complex in European soybean seeds. Mycol. Prog. 19 (5), 455–469. 10.1007/s11557-020-01570-y

[B29] Hu P. Leighton T. Ishkhanova G. Kustu S. (1999). Sensing of nitrogen limitation by *Bacillus subtilis*: Comparison to enteric bacteria. J. Bacteriol. 181 (16), 5042–5050. 10.1128/jb.181.16.5042-5050.1999 10438777 PMC93994

[B30] Hughes C. S. Foehr S. Garfield D. A. Furlong E. E. Steinmetz L. M. Krijgsveld J. (2014). Ultrasensitive proteome analysis using paramagnetic bead technology. Mol. Syst. Biol. 10 (10), 757. 10.15252/MSB.20145625 25358341 PMC4299378

[B31] Joo H. S. Fu C. I. Otto M. (2016). Bacterial strategies of resistance to antimicrobial peptides. Philosophical Trans. R. Soc. B Biol. Sci. 371 (1695), 20150292. 10.1098/rstb.2015.0292 PMC4874390 27160595

[B32] Juhaniewicz-Dębin J. Lasek R. Tymecka D. Burdach K. Bartosik D. Sęk S. (2020). Physicochemical and biological characterization of novel membrane-active cationic lipopeptides with antimicrobial properties. Langmuir 36, 12900–12910. 10.1021/acs.langmuir.0c02135 33085895 PMC7660941

[B33] Kalxdorf M. Müller T. Stegle O. Krijgsveld J. (2021). IceR improves proteome coverage and data completeness in global and single-cell proteomics. Nat. Commun. 12 (1), 4787–4815. 10.1038/s41467-021-25077-6 34373457 PMC8352929

[B34] Klausmann P. Hennemann K. Hoffmann M. Treinen C. Aschern M. Lilge L. (2021). *Bacillus subtilis* high cell density fermentation using a sporulation-deficient strain for the production of surfactin. Appl. Microbiol. Biotechnol. 105, 4141–4151. 10.1007/s00253-021-11330-x 33991199 PMC8140969

[B36] Kulimushi P. Z. Arias A. A. Franzil L. Steels S. Ongena M. (2017). Stimulation of fengycin-type antifungal lipopeptides in *Bacillus amyloliquefaciens* in the presence of the maize fungal pathogen *Rhizomucor variabilis* . Front. Microbiol. 8, 850. 10.3389/fmicb.2017.00850 28555132 PMC5430075

[B37] Li B. Li Q. Xu Z. Zhang N. Shen Q. Zhang R. (2014). Responses of beneficial *Bacillus amyloliquefaciens* SQR9 to different soilborne fungal pathogens through the alteration of antifungal compounds production. Front. Microbiol. 5, 636. 10.3389/fmicb.2014.00636 25484880 PMC4240174

[B38] Lilge L. Ersig N. Hubel P. Aschern M. Pillai E. Klausmann P. (2022). Surfactin shows relatively low antimicrobial activity against *Bacillus subtilis* and other bacterial model organisms in the absence of synergistic metabolites. Microorganisms 10 (4), 779. 10.3390/microorganisms10040779 35456828 PMC9030240

[B39] Lilge L. Vahidinasab M. Adiek I. Becker P. Kuppusamy Nesamani C. Treinen C. (2021). Expression of *degQ* gene and its effect on lipopeptide production as well as formation of secretory proteases in *Bacillus subtilis* strains. MicrobiologyOpen 10 (5), 1241. 10.1002/MBO3.1241 PMC8515880 34713601

[B40] Lin T. P. Chen C. L. Fu H. C. We C. W. Lin G. H. Huang S. H. (2005). Functional analysis of fengycin synthetase FenD. Biochimica Biophysica Acta – Gene Struct. Expr. 1730 (2), 159–164. 10.1016/j.bbaexp.2005.02.005 16102594

[B41] Maget-Dana R. Ptak M. (1995). Interactions of surfactin with membrane models. Biophysical J. 68 (5), 1937–1943. 10.1016/S0006-3495(95)80370-X PMC1282096 7612835

[B42] Mahmood M. (1972). Trace elements for growth and bulbiformin production by *Bacillus subtilis* . J. Appl. Bacteriol. 35 (1), 1–5. 10.1111/j.1365-2672.1972.tb03668.x 4623605

[B43] Malina A. Shai Y. (2005). Conjugation of fatty acids with different lengths modulates the antibacterial and antifungal activity of a cationic biologically inactive peptide. Biochem. J. 390, 695–702. 10.1042/BJ20050520 15907192 PMC1199663

[B45] Meena K. R. Kanwar S. S. (2015). Lipopeptides as the antifungal and antibacterial agents: Applications in food safety and therapeutics. BioMed Res. Int. 2015, 1–9. 10.1155/2015/473050 PMC4303012 25632392

[B47] Oleńska E. Małek W. Wójcik M. Swiecicka I. Thijs S. Vangronsveld J. (2020). Beneficial features of plant growth-promoting rhizobacteria for improving plant growth and health in challenging conditions: A methodical review. Sci. Total Environ. 743, 140682. 10.1016/J.SCITOTENV.2020.140682 32758827

[B71] Oliveras À. Moll L. Riesco-Llach G. Tolosa-Canudas A. Gil-Caballero S. Badosa E. (2021). D-amino acid-containing lipopeptides derived from the lead peptide BP100 with activity against plant pathogens. Int. J. Mol. Sci. 22 (12), 6631. 10.3390/ijms22126631 34205705 PMC8233901

[B48] Olsen J. v. de Godoy L. M. F. Li G. Macek B. Mortensen P. Pesch R. (2005). Parts per million mass accuracy on an orbitrap mass spectrometer via lock mass injection into a C-trap. Mol. Cell. Proteomics 4 (12), 2010–2021. 10.1074/mcp.T500030-MCP200 16249172

[B49] Ongena M. Jacques P. (2008). *Bacillus* lipopeptides: Versatile weapons for plant disease biocontrol. Trends Microbiol. 16 (3), 115–125. 10.1016/J.TIM.2007.12.009 18289856

[B51] Perez-Riverol Y. Bai J. Bandla C. García-Seisdedos D. Hewapathirana S. Kamatchinathan S. (2022). The PRIDE database resources in 2022: A hub for mass spectrometry-based proteomics evidences. Nucleic Acids Res. 50 (D1), D543–D552. 10.1093/nar/gkab1038 34723319 PMC8728295

[B52] Płaza G. Chojniak J. Rudnicka K. Paraszkiewicz K. Bernat P. (2015). Detection of biosurfactants in *Bacillus* species: Genes and products identification. J. Appl. Microbiol. 119 (4), 1023–1034. 10.1111/jam.12893 26171834

[B53] Pršić J. Ongena M. (2020). Elicitors of plant immunity triggered by beneficial bacteria. Front. Plant Sci. 11, 594530. 10.3389/FPLS.2020.594530 33304371 PMC7693457

[B54] Roche B. Aussel L. Ezraty L. Mandin P. Py B. Barras F. (2013). Iron/sulfur proteins biogenesis in prokaryotes: Formation, regulation and diversity. Biochimica Biophysica Acta – Bioenergetics 1827 (3), 455–469. 10.1016/j.bbabio.2012.12.010 23298813

[B55] Romero D. De Vicente A. Rakotoaly R. H. Dufour S. E. Veening J. W. Arrebola E. (2007). The iturin and fengycin families of lipopeptides are key factors in antagonism of *Bacillus subtilis* toward *Podosphaera fusca* . Mol. Plant-Microbe Interact. MPMI 20 (4), 430–440. 10.1094/MPMI-20-4-0430 17427813

[B56] Steinke K. Mohite O. S. Weber T. Kovács Á. T. (2021). Phylogenetic distribution of secondary metabolites in the *Bacillus subtilis* species complex. MSystems 6 (2), 000577–e121. 10.1128/mSystems.00057-21 PMC8546965 33688015

[B58] Summerell B. A. Salleh B. Leslie J. F. (2007). A utilitarian approach to *Fusarium* identification. Plant Dis. 87 (2), 117–128. 10.1094/PDIS.2003.87.2.117 30812915

[B59] Tabbene O. Kalai L. Ben Slimene I. Karkouch I. Elkahoui S. Gharbi A. (2011). Anti-*Candida* effect of bacillomycin D-like lipopeptides from *Bacillus subtilis* B38. FEMS Microbiol. Lett. 316 (2), 108–114. 10.1111/J.1574-6968.2010.02199.X 21204933

[B60] Tang Q. Bie X. Lu Z. Lv F. Tao Y. Qu X. (2014). Effects of fengycin from *Bacillus subtilis* fmbJ on apoptosis and necrosis in *Rhizopus stolonifer* . J. Microbiol. 52 (8), 675–680. 10.1007/S12275-014-3605-3 25098563

[B61] Théatre A. Cano-Prieto C. Bartolini M. Laurin Y. Deleu M. Niehren J. (2021). The surfactin-like lipopeptides from *Bacillus* spp.: Natural biodiversity and synthetic Biology for a broader application range. Front. Bioeng. Biotechnol. 9, 623701. 10.3389/fbioe.2021.623701 33738277 PMC7960918

[B62] Tosato V. Albertini A. M. Zotti M. Sonda S. Bruschi C. V. (1997). Sequence completion, identification and definition of the fengycin operon in *Bacillus subtilis* 168. Microbiology 143 (11), 3443–3450. 10.1099/00221287-143-11-3443 9387222

[B63] Tsotetsi T. Nephali L. Malebe M. Tugizimana F. (2022). *Bacillus* for plant growth promotion and stress resilience: What have we learned? Plants 11 (19), 2482. 10.3390/PLANTS11192482 36235347 PMC9571655

[B64] Tyanova S. Temu T. Sinitcyn P. Carlson A. Hein M. Y. Geiger T. (2016). The Perseus computational platform for comprehensive analysis of (prote)omics data. Nat. Methods 13 (9), 731–740. 10.1038/nmeth.3901 27348712

[B66] Vahidinasab M. Adiek I. Hosseini B. Akintayo S. O. Abrishamchi B. Pfannstiel J. (2022). Characterization of *Bacillus velezensis* UTB96, demonstrating improved lipopeptide production compared to the strain *B. velezensis* FZB42. Microorganisms 10 (11), 2225. 10.3390/microorganisms10112225 36363818 PMC9693074

[B68] Walsh C. T. (2014). Blurring the lines between ribosomal and nonribosomal peptide scaffolds. ACS Chem. Biol. 9 (8), 1653–1661. 10.1021/CB5003587 24883916

[B69] Willenbacher J. Yeremchuk W. Mohr T. Syldatk C. Hausmann R. (2015). Enhancement of Surfactin yield by improving the medium composition and fermentation process. Amb. Express 5 (1), 57–59. 10.1186/s13568-015-0145-0 26297438 PMC4546119

