# Supplementary material for: Characterization ofantifungal properties of lipopeptide-producing Bacillus velezensis strains and their proteome-based response to the phytopathogens, Diaporthe spp
Source: Front Bioeng Biotechnol. 2023 Aug 7;11:1228386. doi: 10.3389/fbioe.2023.1228386 (PMC10440741; doi:10.3389/fbioe.2023.1228386)
Supplement: Supplementary file 1 [file DataSheet1.PDF]

Supplementary Material

Characterization of antifungal properties of lipopeptide-producing *Bacillus velezensis* strains and their proteome-based response to the phytopathogens, *Diaporthe* spp.

Stephen Olusanmi Akintayo[1], Behnoush Hosseini[2], Maliheh Vahidinasab[1], Marc Messmer[1], Jens Pfannstiel[3], Ute Bertsche[3], Philipp Hubel[3] Marius Henkel[4], Rudolf Hausmann[1], Ralf T. Voegele[2], Lars Lilge[1,5]*

*** Correspondence:** Lars Lilge: lars.lilge@uni-hohenheim.de

Table S1: Bacterial and fungal strains used in this study.

Strain	Sources and references
**Bacteria**	
B. velezensis ES1-02	(Akintayo et al., 2022)
B. velezensis EFSO2-04	(Akintayo et al., 2022)
B. velezensis FZB42	Lab stock; (Krebs et al., 1998)
B. velezensis QST713	Lab stock; (Chen et al., 2007)
**Fungi**	
D. caulivora (DPC_HOH2)	(Hosseini et al., 2020)
D. eres (DPC_HOH7)	(Hosseini et al., 2020)
D. novem (DPC_HOH15)	(Hosseini et al., 2020)
D. longicolla (DPC_HOH20)	(Hosseini et al., 2020)

Table S2: List of primers used in this study.

Gene	Forward Primer (5'-3')	Reverse Primer (5'-3')	References
srfAA	GTGCTTGAATATAACACCGC	CGTATTTGGCGTTTTATCATC	This study
ituB	CACGAACAGACAAAACA	*ituB* (iturin A)<br>TGCGCAAAGCATCGT<br>*ituB* (bacillomycin D)<br>CTTGCGGCGTTTGTG<br>*ituB* (bacillomycin L)<br>GGTCGCTCCTGAATCT	Dunlap et al., 2019<br>Dunlap et al., 2019<br>Dunlap et al., 2019
fenA	CACAAGTATCTGCAATCTCG	CATAAACAGAGAGATGATGCTG	This study
fenD	CAGGATATTTATCCGCTGTC	CATACACGCTTGAATCGTATC	This study

Table S3: Identification of LP-encoding genes by PCR approach.

Strain	Surfactin	Iturin family of LP (*ituB*)	Fengycin
ES1-02	*srfAA*	Bacillomycin L	*fenA*
EFSO2-04	*srfAA*	Bacillomycin L	*fenA*
QST713	*srfAA*	Iturin A	*fenA, fenD*
FZB42	*srfAA*	Bacillomycin D	*fenA, fenD*

Table S4: Number of quantified protein groups by MaxQuant 2.0.1.0 and after re-quantification with IceR.

Sample	MaxQuant 2.0.1.0	IceR
	Number of quantified protein groups	Number of quantified protein groups
S1	1084	1236
S1c	997	1242
S2	810	1235
S2c	993	1237

S3	1085	1239
S3c	1012	1232
S4	898	1234
S4c	1128	1238
S5	1063	1240
S5c	1048	1236
S6	1034	1236
S6c	1157	1239
S7	1103	1238
S7c	1141	1239

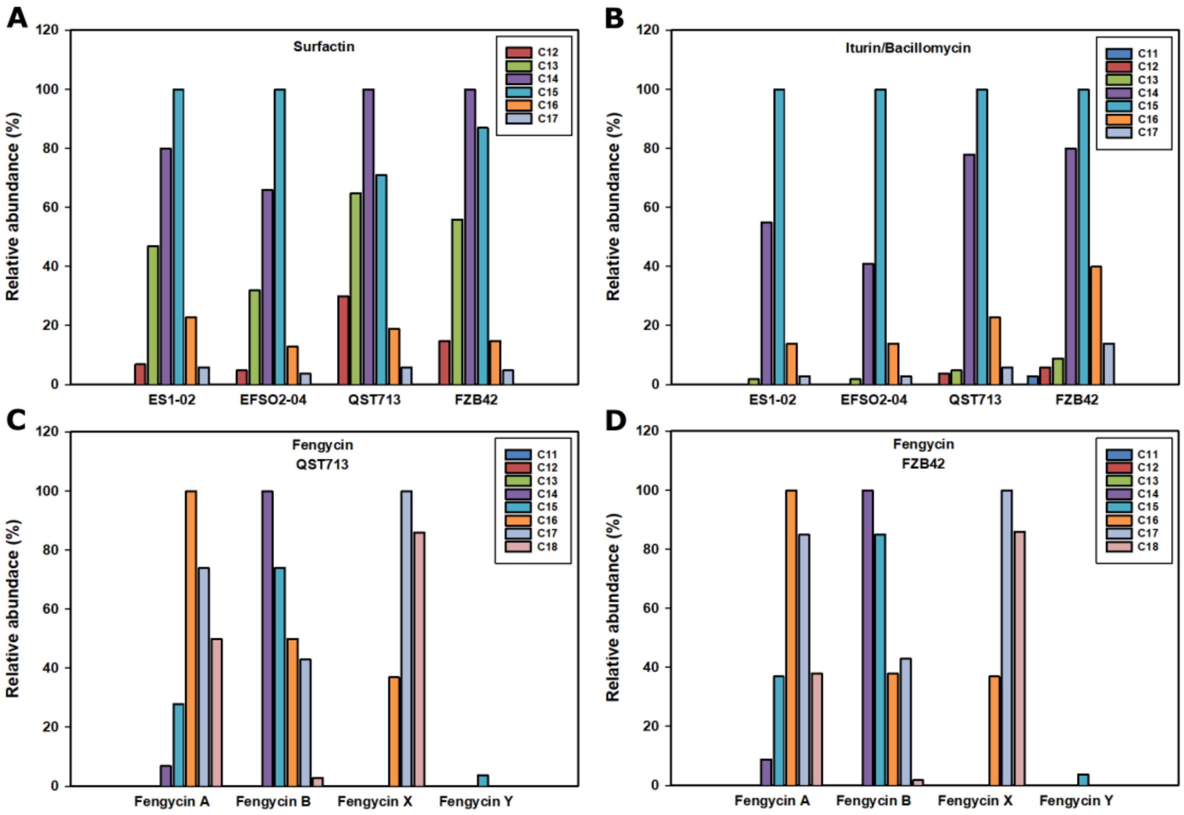

Figure S1: Relative abundance of isoforms of surfactin (A), iturin/bacillomycin (B) and fengycin (C, D) in *B. velezensis* strains ES1-02 (A, B), EFSO2-04 (A, B), QST713 (A-D) and FZB42 (A-D).

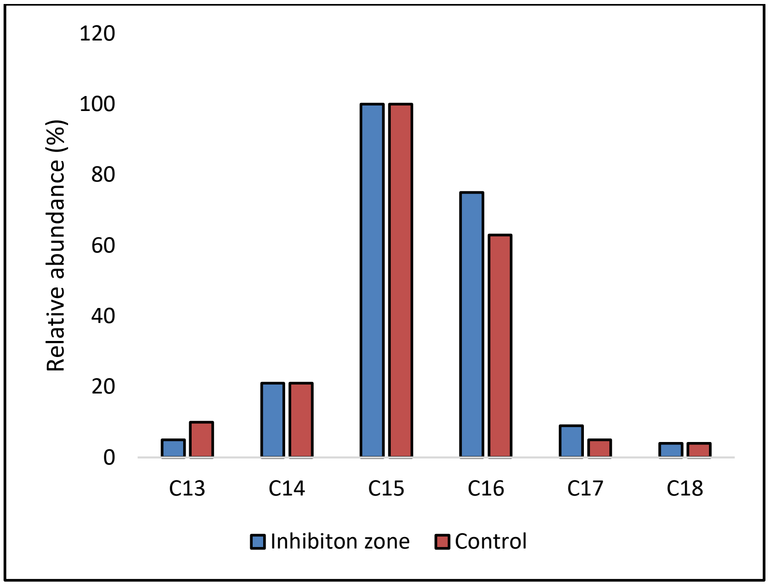

Figure S2: Relative abundance of surfactin isoforms determined in the zone of inhibition between *B. velezensis* strains ES1-02 co-cultured in presence (blue-inhibition zone) or absence of *D. longicolla* DPC_HOH20 (orange-control).

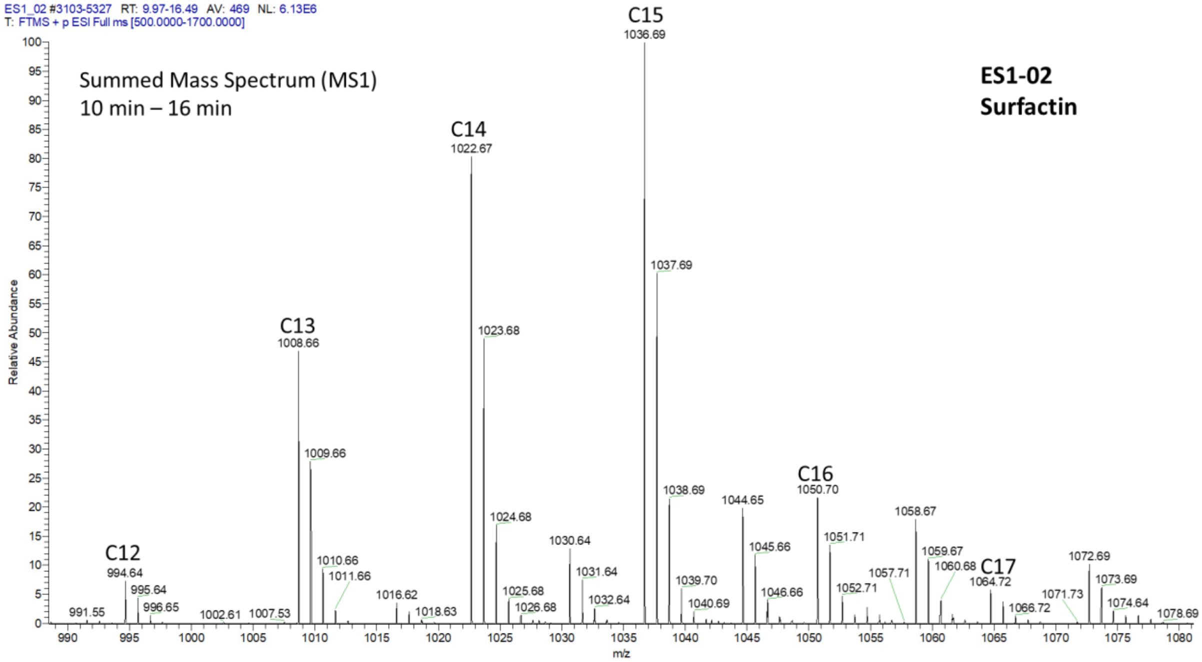

Figure S3: MS spectrum of surfactin produced by *B. velezensis* ES1-02.

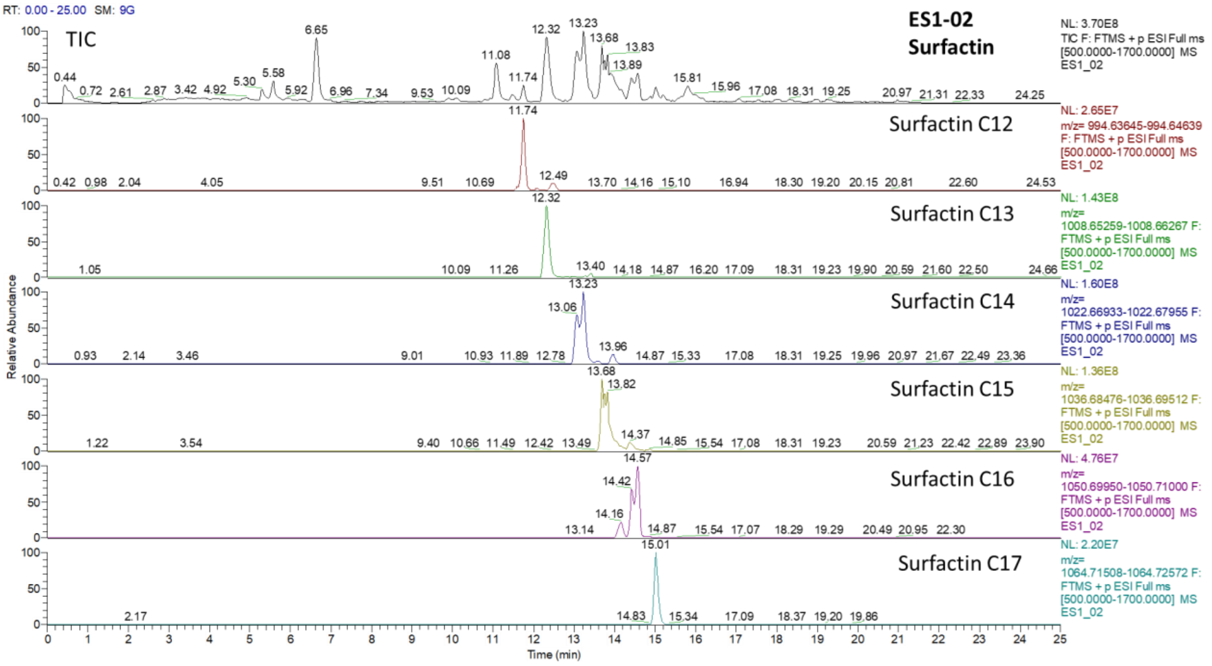

Figure S4: Extracted ion chromatogram of surfactin produced by *B. velezensis* ES1-02.

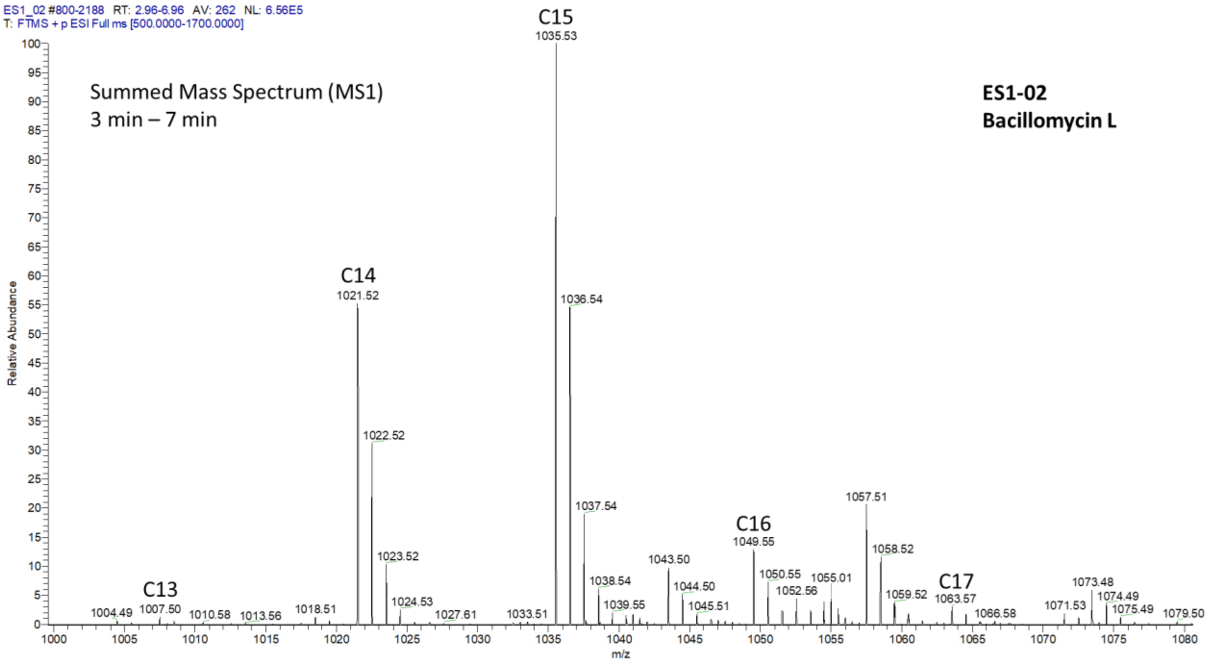

Figure S5: MS spectrum of bacillomycin L produced by *B. velezensis* ES1-02.

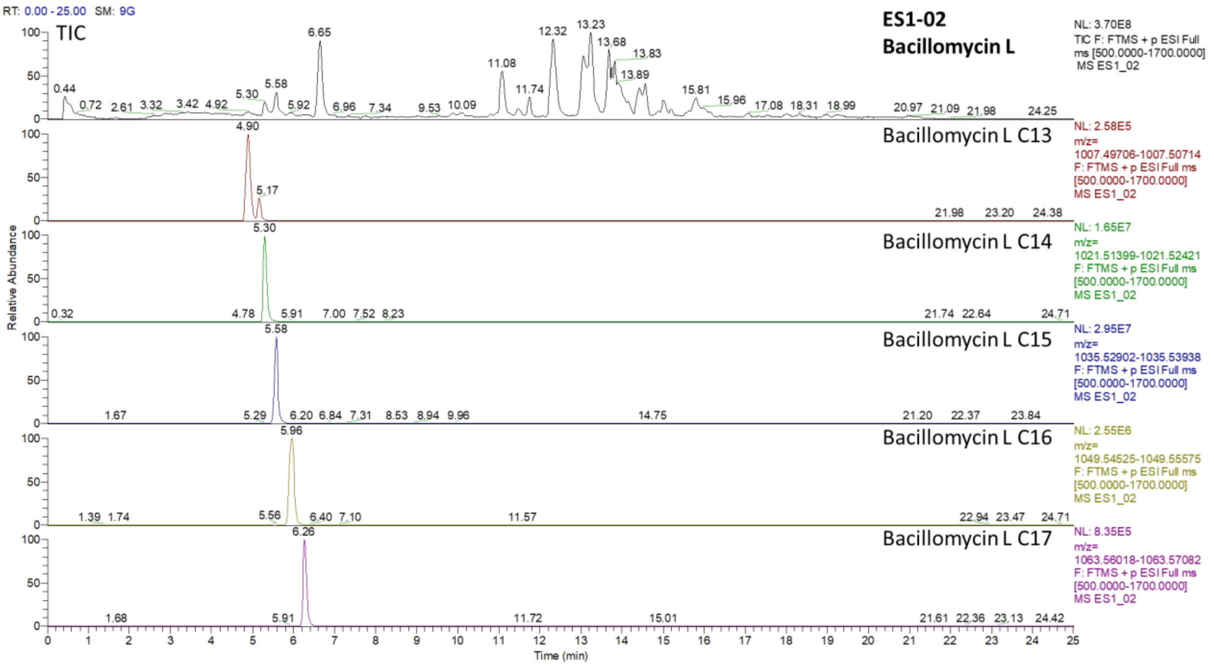

Figure S6: Extracted ion chromatogram of bacillomycin L produced by *B. velezensis* ES1-02.

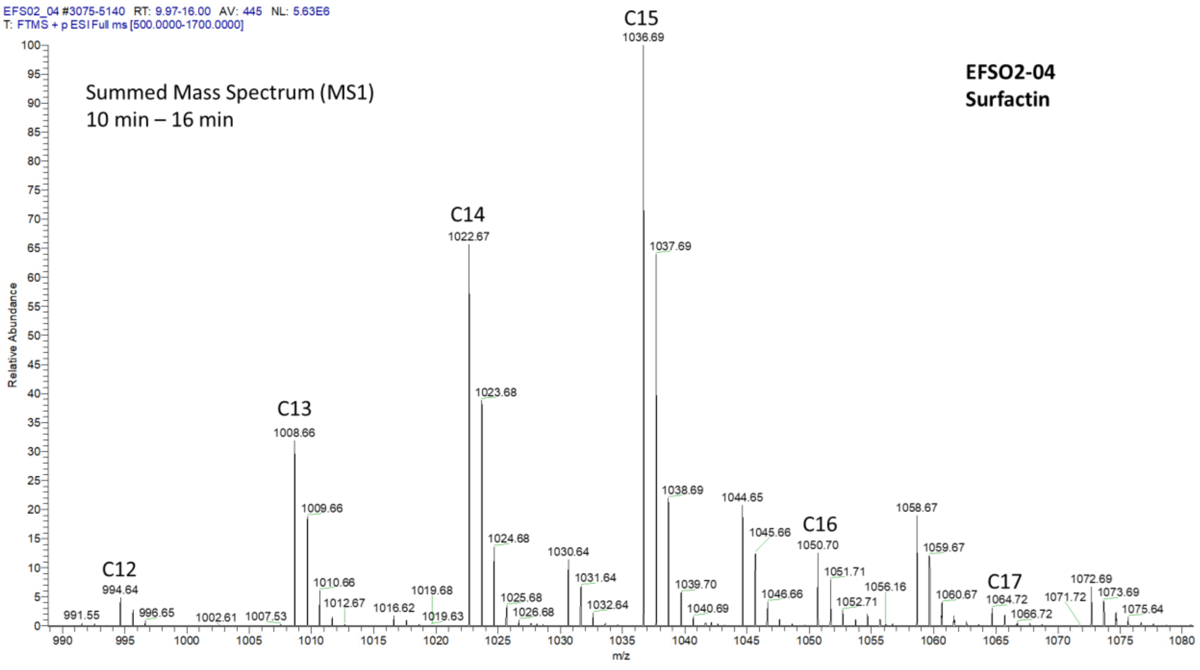

Figure S7: MS spectrum of the surfactin produced by *B. velezensis* EFSO2-04.

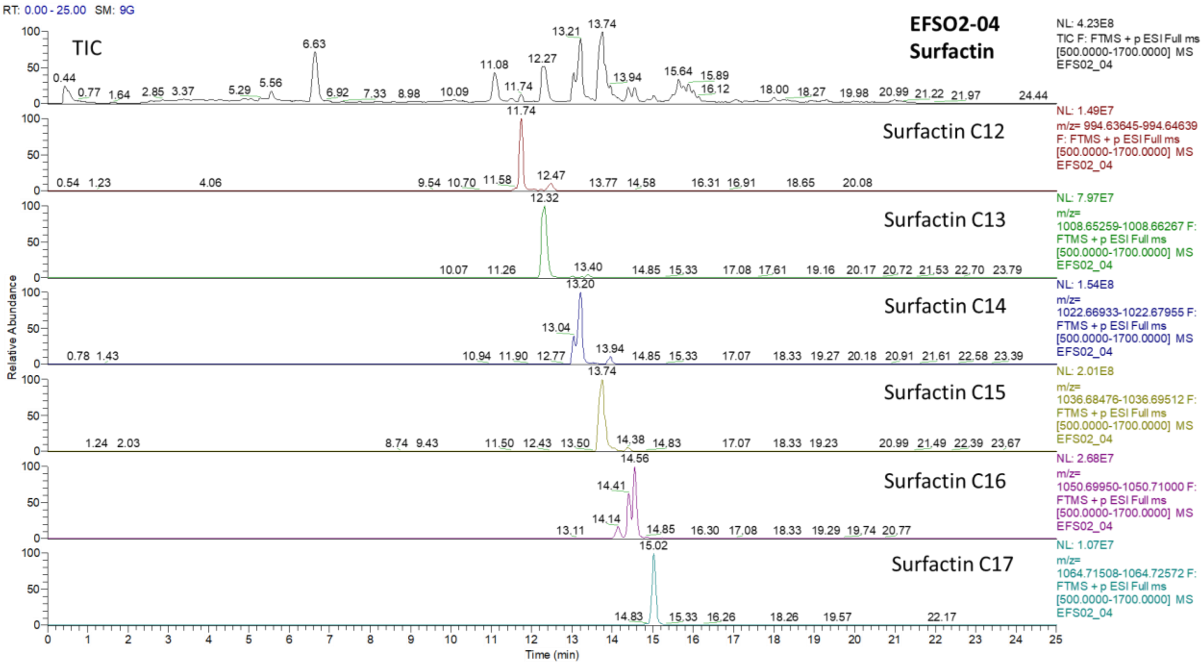

Figure S8: Extracted ion chromatogram of surfactin produced by *B. velezensis* EFSO2-04.

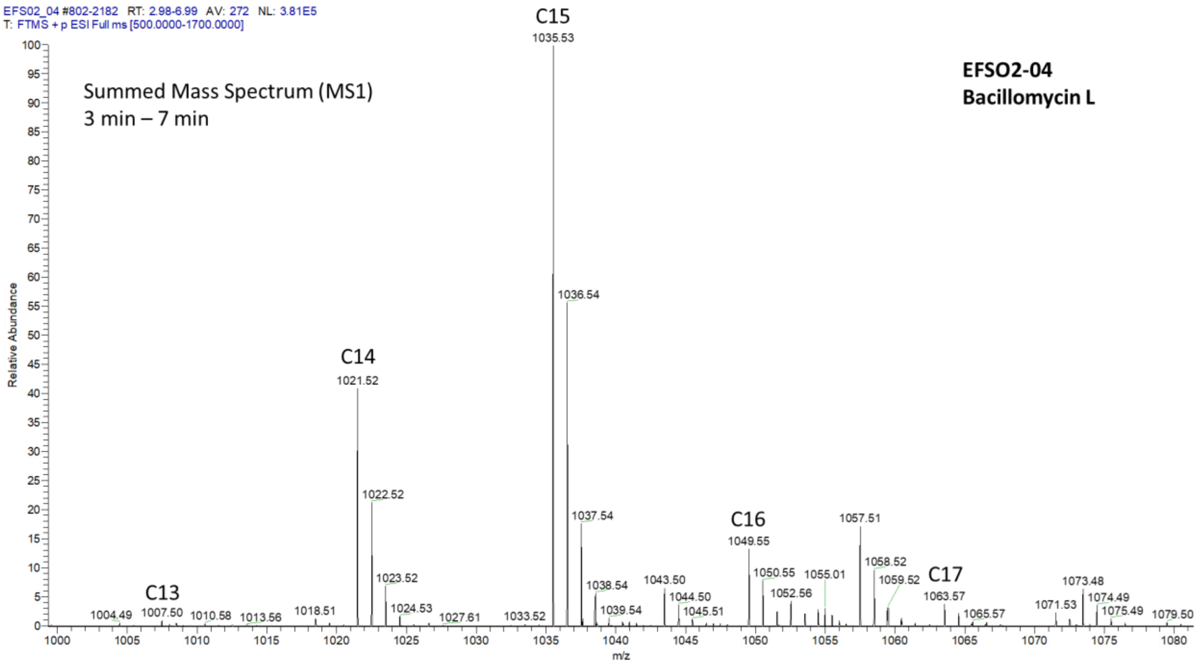

Figure S9: MS spectrum of the bacillomycin L produced by *B. velezensis* ES1-02.

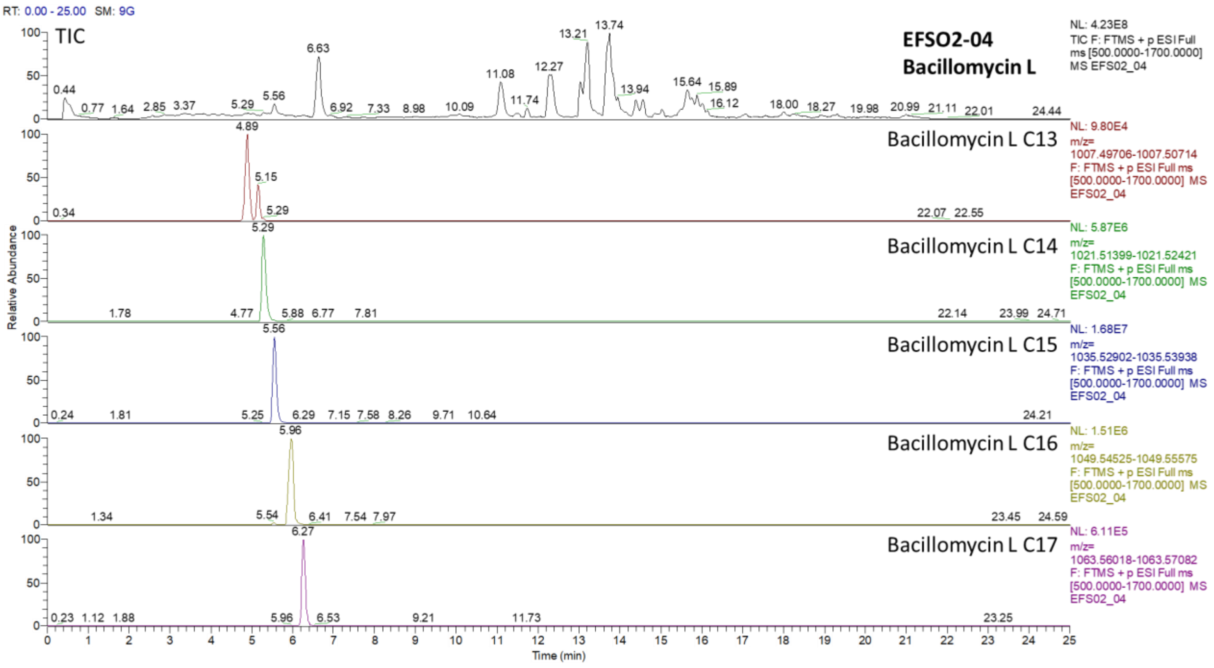

Figure S10: Extracted ion chromatogram of bacillomycin L produced by *B. velezensis* EFSO2-04.

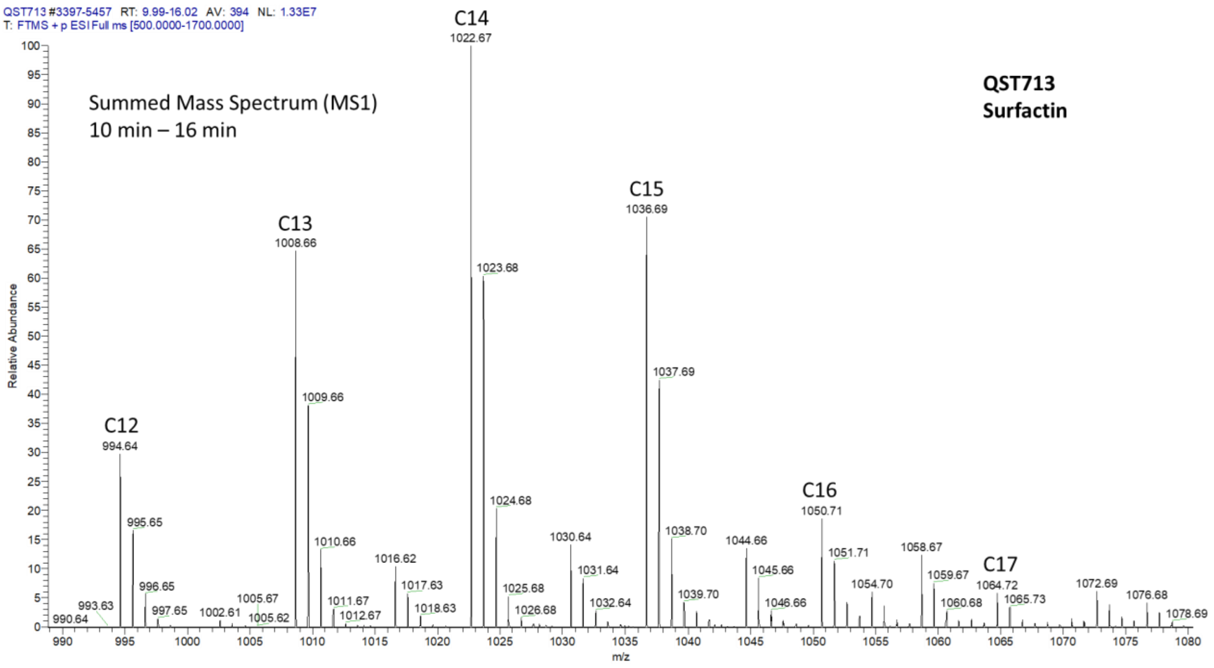

Figure S11: MS spectrum of the surfactin produced by *B. velezensis* QST713.

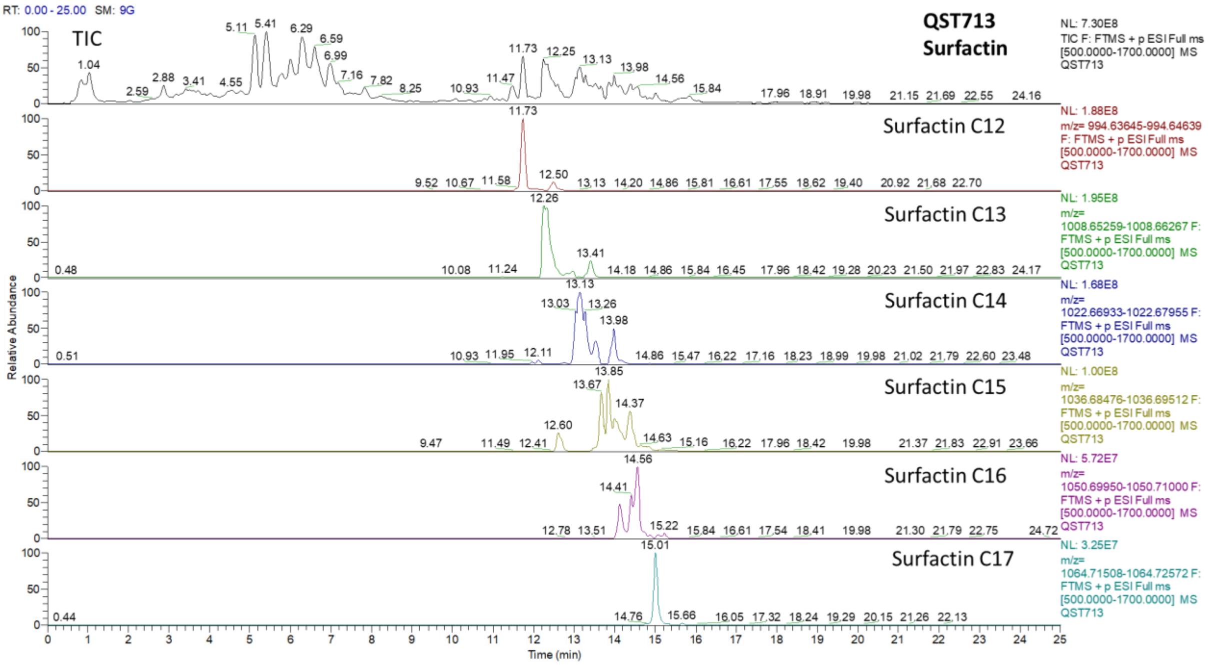

Figure S12: Extracted ion chromatogram of surfactin produced by *B. velezensis* QST713.

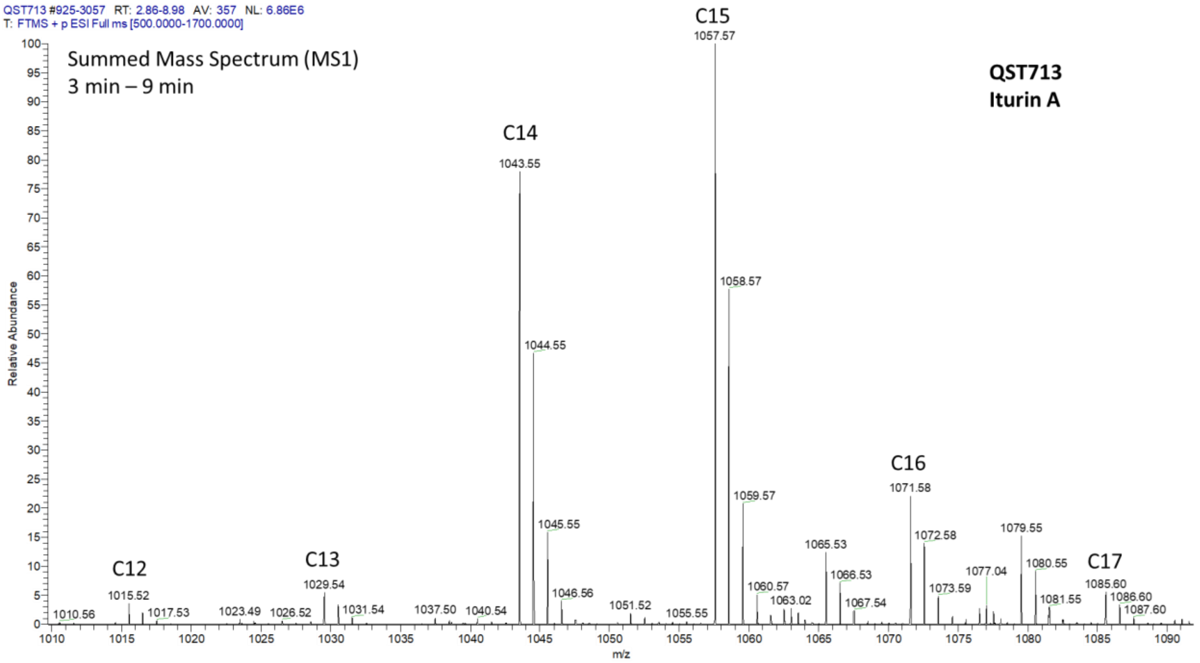

Figure S13: MS spectrum of the iturin A produced by *B. velezensis* QS713.

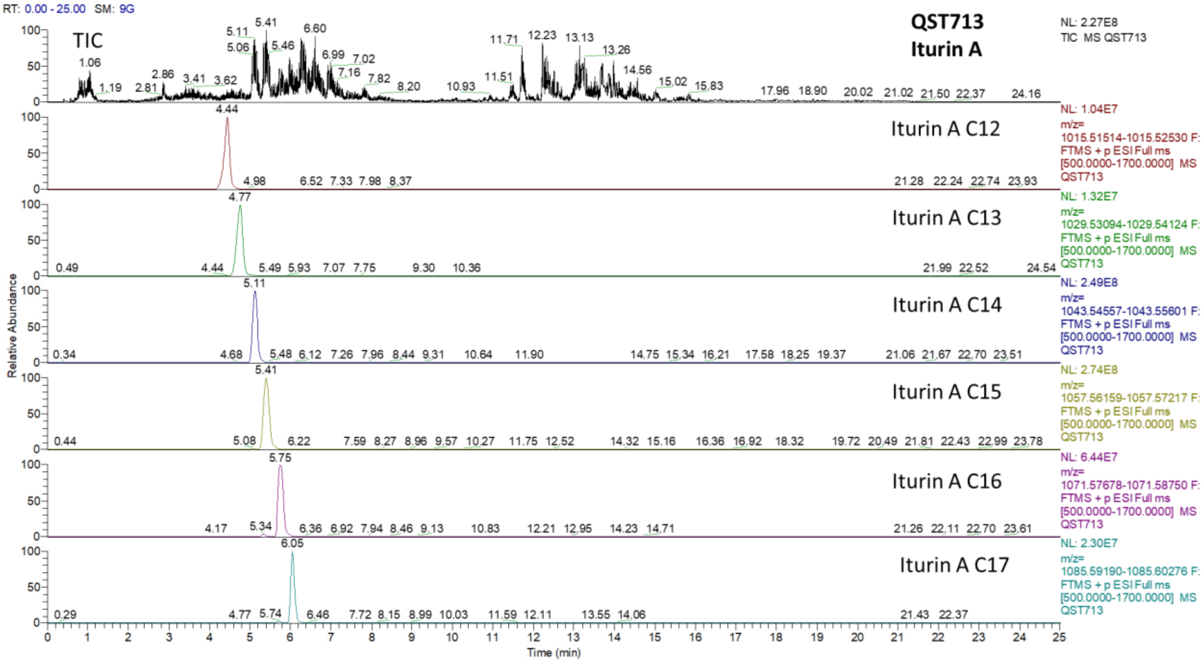

Figure S14: Extracted ion chromatogram of iturin A produced by *B. velezensis* QST713.

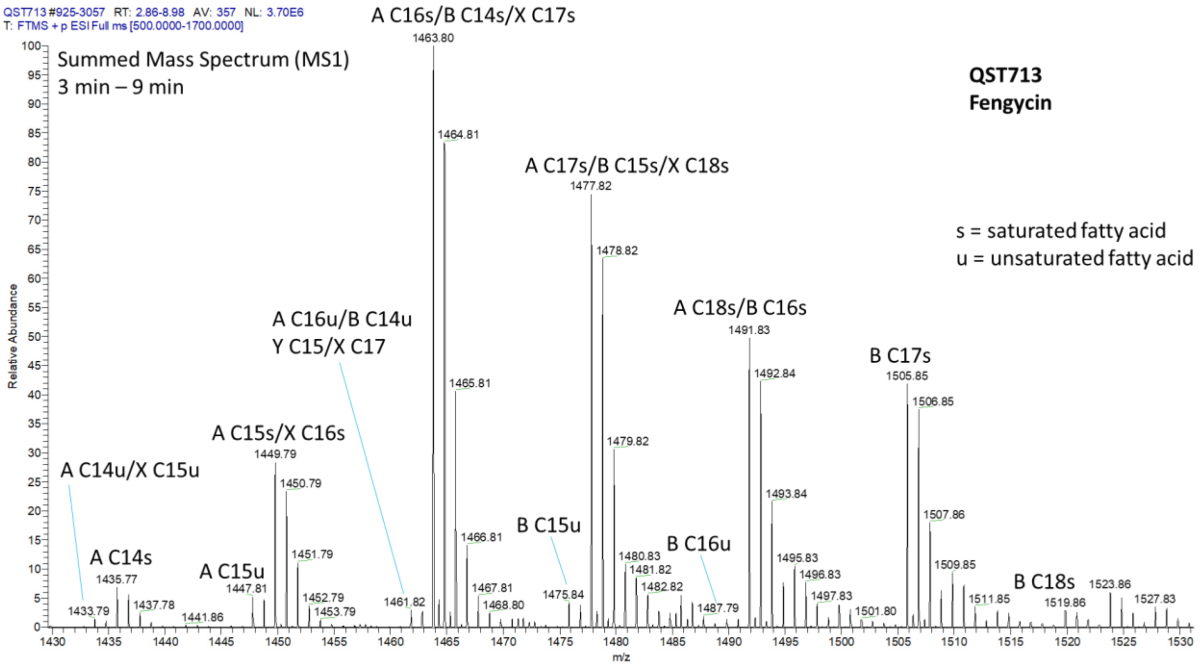

Figure S15: MS spectrum of the fengycin produced by *B. velezensis* QST713.

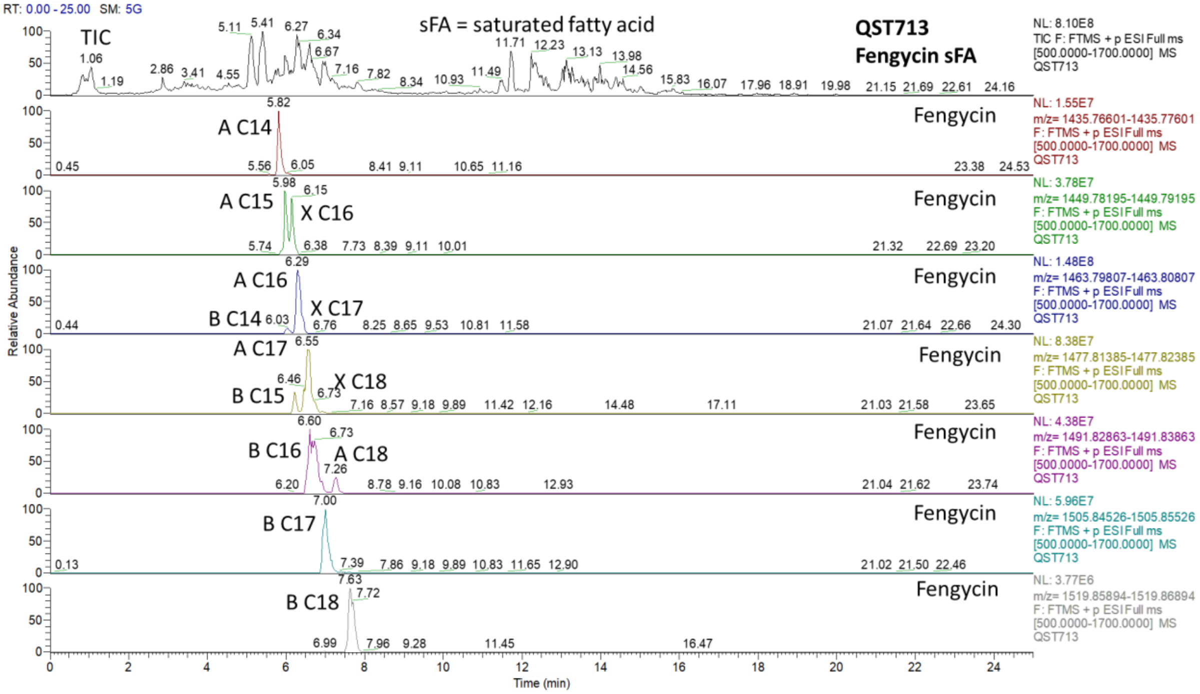

Figure S16: Extracted ion chromatogram of fengycin (saturated fatty acid) produced by *B. velezensis* QST713.

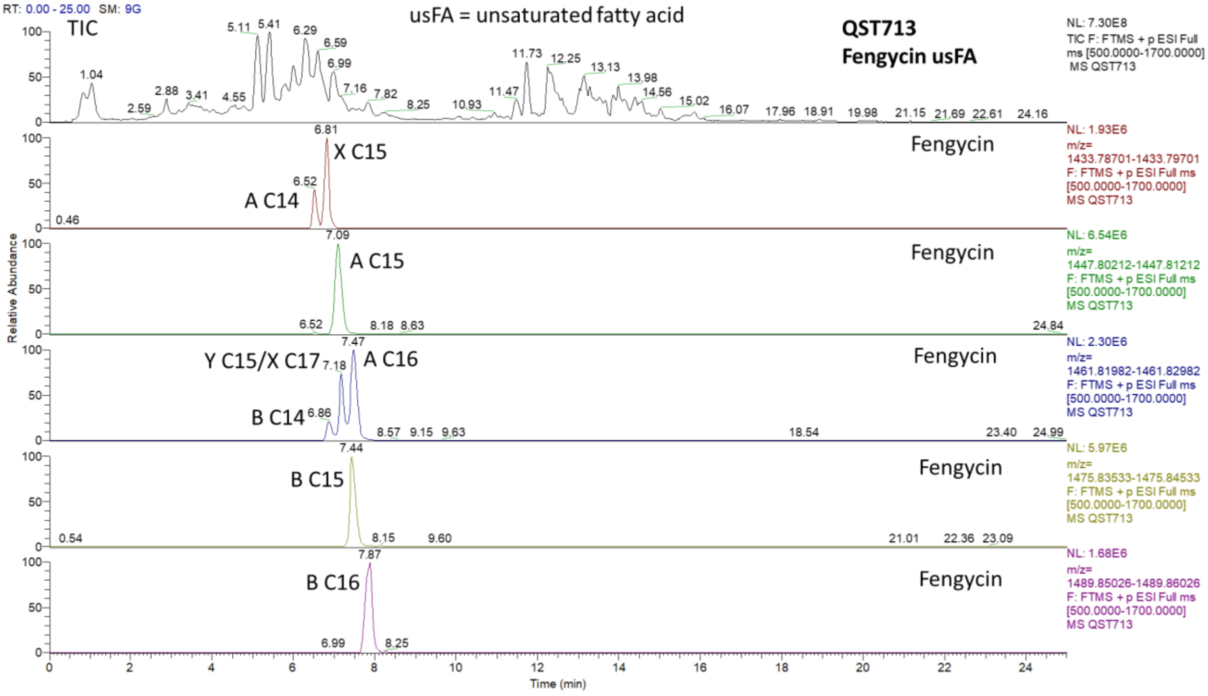

Figure S17: Extracted ion chromatogram of fengycin (unsaturated fatty acid) produced by *B. velezensis* QST713.

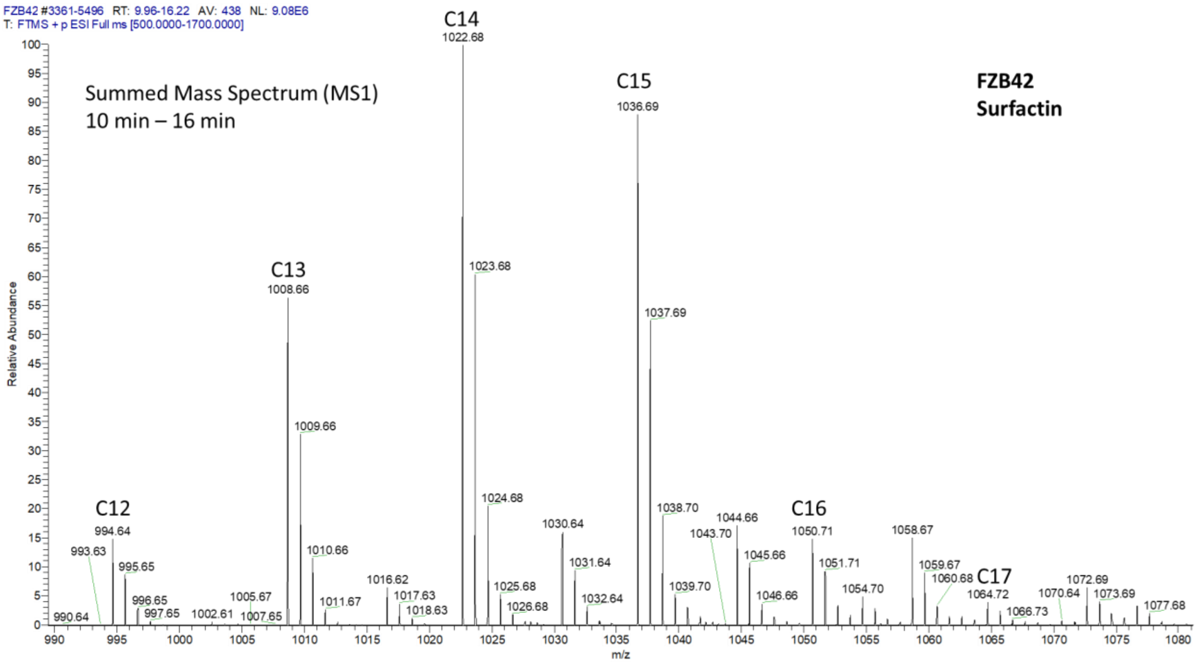

Figure S18: MS spectrum of the surfactin produced by *B. velezensis* FZB42.

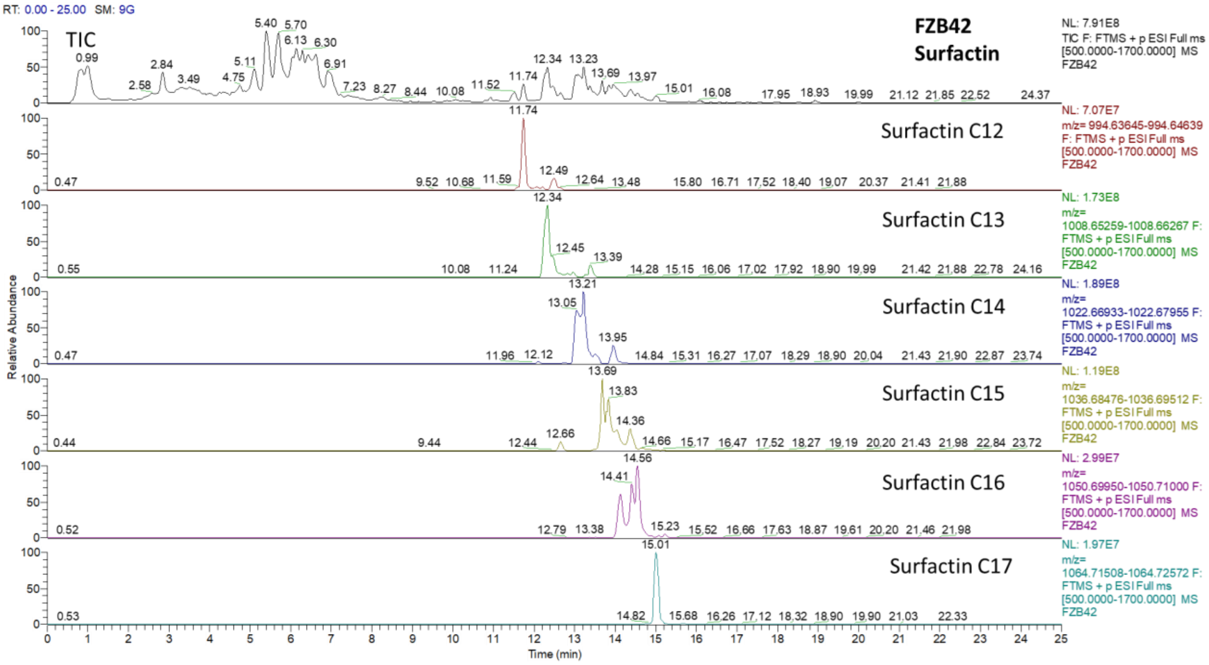

Figure S19: Extracted ion chromatogram of surfactin produced by *B. velezensis* FZB42.

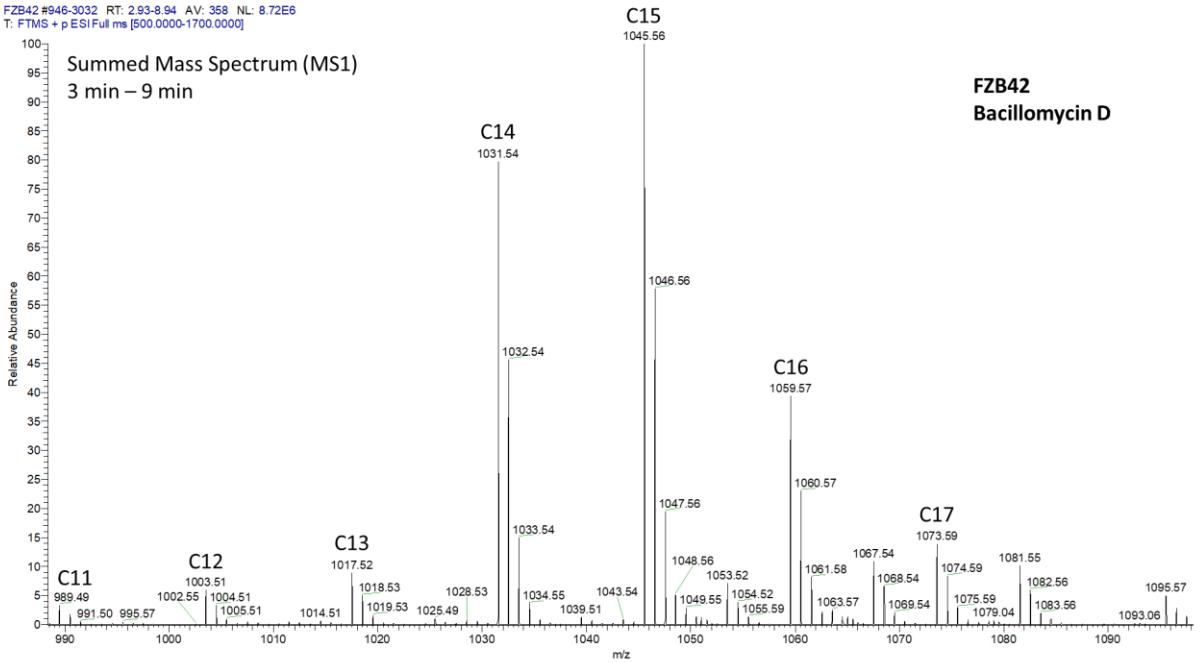

Figure S20: MS spectrum of the bacillomycin D produced by *B. velezensis* FZB42.

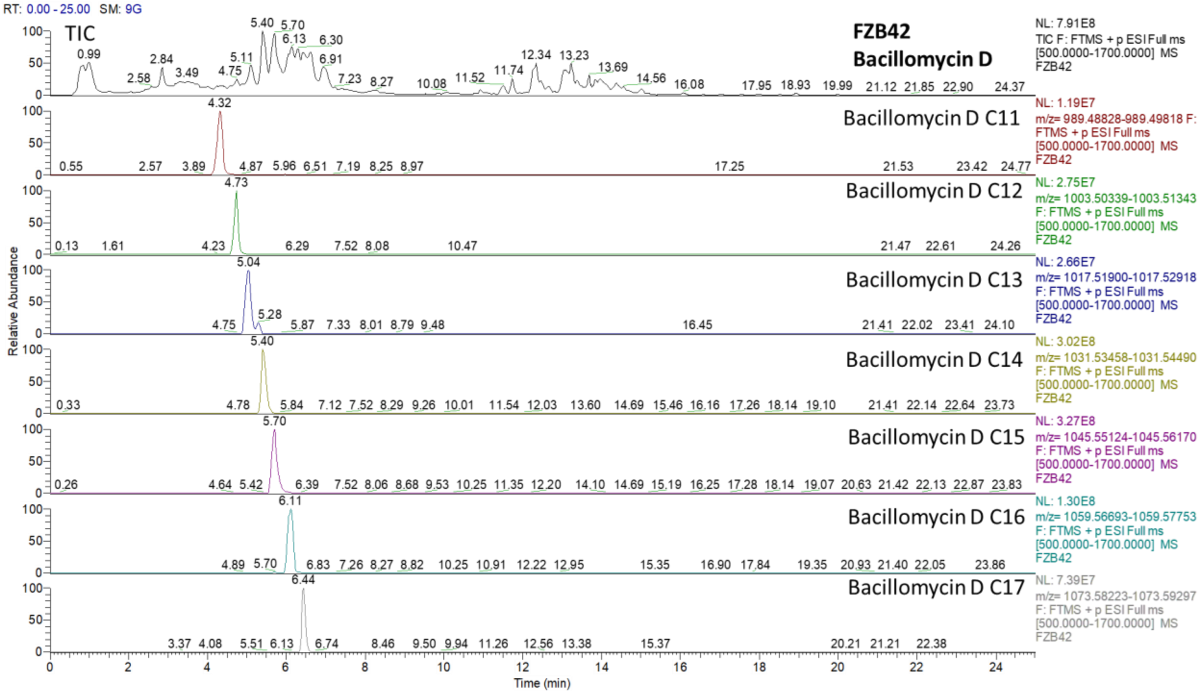

Figure S21: Extracted ion chromatogram of bacillomycin produced by *B. velezensis* FZB42.

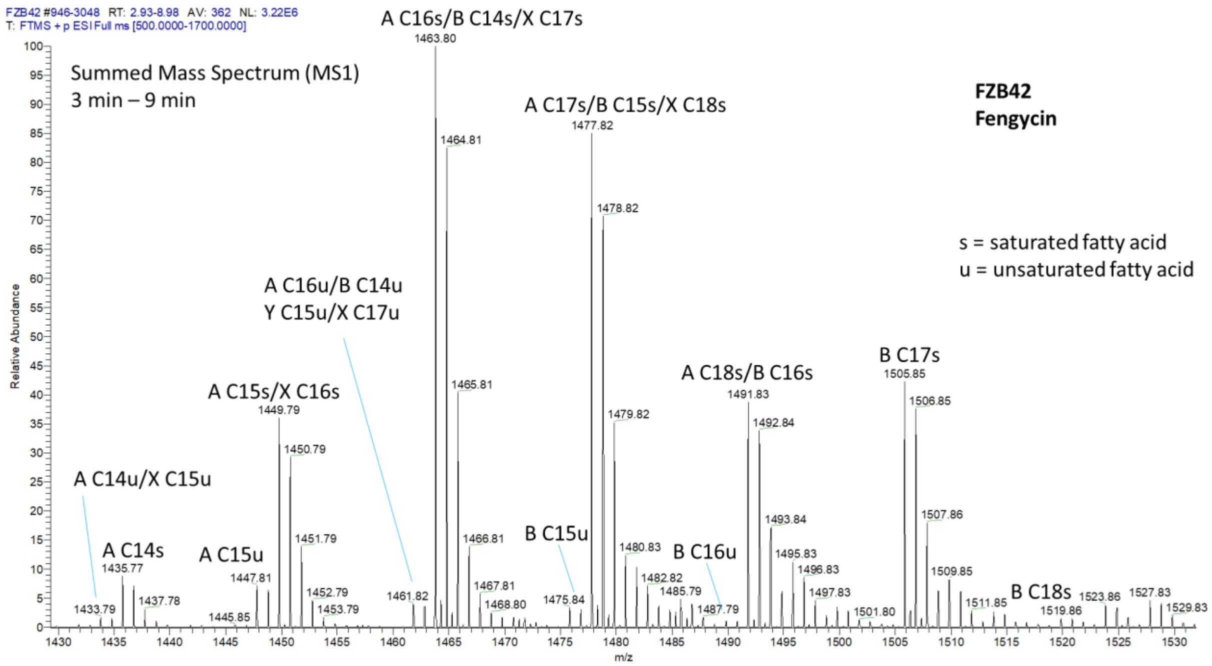

Figure S22: MS spectrum of the fengycin produced by *B. velezensis* FZB42.

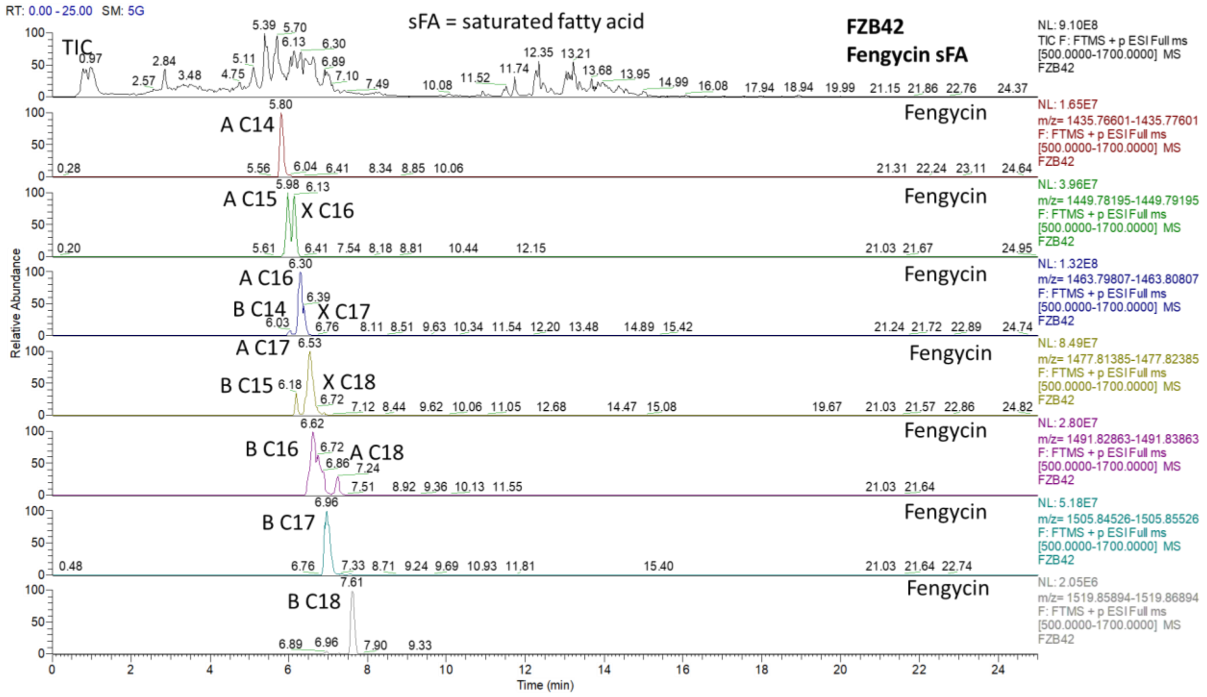

Figure S23: Extracted ion chromatogram of fengycin (saturated fatty acid) produced by *B. velezensis* FZB42.

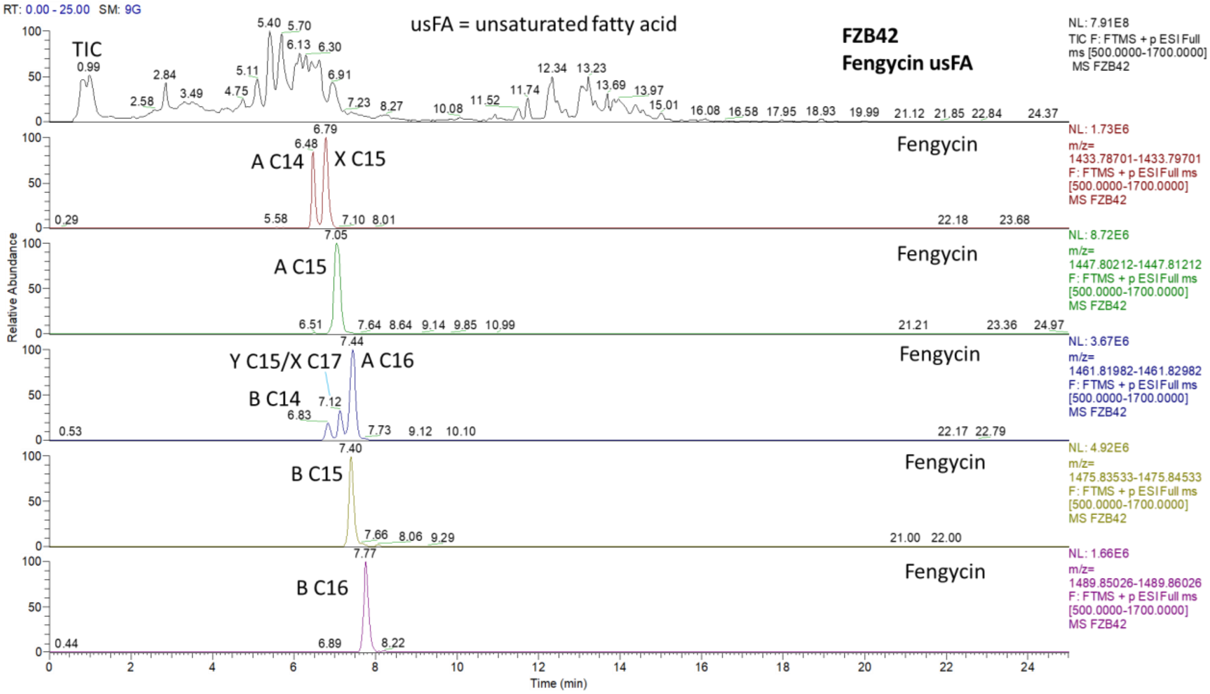

Figure S24: Extracted ion chromatogram of fengycin (unsaturated fatty acid) produced by *B. velezensis* FZB42.
